# haploMAGIC: accurate phasing and detection of recombination in multiparental populations despite genotyping errors

**DOI:** 10.1093/g3journal/jkae109

**Published:** 2024-05-29

**Authors:** Jose A Montero-Tena, Nayyer Abdollahi Sisi, Tobias Kox, Amine Abbadi, Rod J Snowdon, Agnieszka A Golicz

**Affiliations:** Department of Agrobioinformatics, IFZ Research Center for Biosystems, Land Use and Nutrition, Justus Liebig University, Heinrich Buff Ring 26, 35392 Giessen, Germany; Department of Plant Breeding, IFZ Research Center for Biosystems, Land Use and Nutrition, Justus Liebig University, Heinrich Buff Ring 26, 35392 Giessen, Germany; NPZ Innovation GmbH, Hohenlieth-Hof, 24363 Holtsee, Germany; NPZ Innovation GmbH, Hohenlieth-Hof, 24363 Holtsee, Germany; Department of Plant Breeding, IFZ Research Center for Biosystems, Land Use and Nutrition, Justus Liebig University, Heinrich Buff Ring 26, 35392 Giessen, Germany; Department of Agrobioinformatics, IFZ Research Center for Biosystems, Land Use and Nutrition, Justus Liebig University, Heinrich Buff Ring 26, 35392 Giessen, Germany

**Keywords:** oilseed rape, recombination, genotyping, Multiparental Populations, Multiparent Advanced Generation Inter-Cross (MAGIC), MPP

## Abstract

Recombination is a key mechanism in breeding for promoting genetic variability. Multiparental populations (MPPs) constitute an excellent platform for precise genotype phasing, identification of genome-wide crossovers (COs), estimation of recombination frequencies, and construction of recombination maps. Here, we introduce haploMAGIC, a pipeline to detect COs in MPPs with single-nucleotide polymorphism (SNP) data by exploiting the pedigree relationships for accurate genotype phasing and inference of grandparental haplotypes. haploMAGIC applies filtering to prevent false-positive COs due to genotyping errors (GEs), a common problem in high-throughput SNP analysis of complex plant genomes. Hence, it discards haploblocks not reaching a specified minimum number of informative alleles. A performance analysis using populations simulated with AlphaSimR revealed that haploMAGIC improves upon existing methods of CO detection in terms of recall and precision, most notably when GE rates are high. Furthermore, we constructed recombination maps using haploMAGIC with high-resolution genotype data from 2 large multiparental populations of winter rapeseed (*Brassica napus*). The results demonstrate the applicability of the pipeline in real-world scenarios and showed good correlations in recombination frequency compared with alternative software. Therefore, we propose haploMAGIC as an accurate tool at CO detection with MPPs that shows robustness against GEs.

## Introduction

Meiotic recombination refers to the reciprocal exchange of DNA between homologous chromosomes that occurs during gamete formation (Hegde and Crowley 2019). Recombination is a key aspect in breeding for promoting genetic variation by introducing new combinations of alleles that are not present in the parental chromosomes (Bolcun-Filas and Schimenti 2012; Epstein *et al*. 2023). However, the distribution of crossovers (COs) is commonly not homogeneous across the genome but rather concentrated in regions called CO hotspots (Lambing *et al*. 2017). Recombination maps facilitate the identification of CO hotspots by estimating the frequency of recombination between pairs of markers located on the same chromosomes, for example, by exploiting polymorphism data in populations (Qanbari and Wittenburg 2020). Genetic variation can be expanded through plant breeding programs by understanding CO positioning and altering the position and/or the frequency of CO events (Lambing *et al*. 2017). Previous studies about recombination have been conducted in crop species, such as maize (Li, Li *et al*. 2015), tomato (De Haas *et al*. 2017), oilseed rape, or chickpea (Bayer *et al*. 2015).

Multiparental populations (MPPs) consist of fully related individuals with genotyping information descending from a set of founder lines, which are typically homozygous and selected to maximize genetic diversity. MPPs are often used for trait mapping with genotyping data commonly available for founders and the last generation (Scott *et al*. 2020). Genotyping data for the intermediate generations might also be available, allowing for the use of parental and offspring genotypes to infer COs in consecutive generations by phasing family trio data (Williams *et al*. 2010; Miller and Piccolo 2021). This allows for the identification of haplotype blocks, which are regions of DNA inherited on the same homologous chromosome after meiotic recombination. These properties give MPPs high resolution and power, making them excellent platforms for mapping recombination events or quantitative mapping (Li, Yongxiang, *et al*. 2015; Sannemann *et al*. 2015; Descalsota *et al*. 2018; Scott *et al*. 2020).

Some methods, such as duoHMM (O’Connell *et al*. 2014; Al Bkhetan *et al*. 2019) and LINKPHASE3 (Druet and Georges 2015), have shown good performance with related individuals despite not specifically being designed for use with MPPs with full pedigrees. duoHMM (O’Connell *et al*. 2014) is a well-established method that applies a family-based correction of the haplotypes inferred by SHAPEIT (Delaneau *et al*. 2013). LINKPHASE3 is a pedigree-based phasing algorithm specialized in half-sib families, in which offspring might share only 1 parent, reportedly performing better than duoHMM in terms of avoidance of errors in phasing, or CO detection with simulations of half-sib families (Druet and Georges 2015).

Here, we introduce haploMAGIC, a pipeline for phasing and CO detection that is specialized in MPPs with single-nucleotide polymorphism (SNP) genotypes of individuals derived from inbred founder lines. *F*1 scores obtained while detecting recombination events in simulated populations revealed that haploMAGIC outperformed duoHMM and LINKPHASE3 and behaved consistently despite increasing genotyping error (GE) rates. This is due to the 2 customizable filtering options that haploMAGIC implements, namely (i) haploblock filtering by minimum number of informative alleles and (ii) different types of phase imputation of unresolved loci, which allows for the efficient removal of false-positive haploblocks causing false recombination events. We analyzed the effect of filtering on performance and tested haploMAGIC on real-world data. haploMAGIC is available in GitHub, https://github.com/GoliczGenomeLab/haploMAGIC.

## Materials and methods

### Implementation

haploMAGIC requires PLINK (Purcell *et al*. 2007) flat files: a PED file, containing pedigree information and diploid SNP genotypes (Liu *et al*. 2020), and a MAP file, with the genome locations (bp) of the SNP markers. Genetic distances in the MAP file are not used. Additionally, users must provide the arguments regarding filtering options and other functionalities (Table 1).

**Table 1.** haploMAGIC arguments with functions, options, and description of the behavior of each option.

Argument	Function	Option	Description
Minimum number of informative alleles per haploblock (min)	Removes and imputes haploblocks with < min informative alleles. Any min ≥ 1	min = 1	No filter applied
		min = 2	Retains haploblocks that have at least 2 informative alleles
Phase imputation (imp)	Imputes the phase of unresolved loci enabling their use for phasing in following generations	imp = imputeNot	No phase imputation
		imp = imputeTHonly	Only imputes triply heterozygous loci
		imp = imputeAll	Imputes all unresolved loci (Mendelian error, missing data, triply heterozygous)
Postimputation phase correction (cor)	Corrects the phases of triply heterozygous loci	cor = correctNot	No correction. Forced by imp = imputeNot. Not recommended in combination with imp = imputeTHonly or imp = imputeAll
		cor = correctFalseHom	Undoes phases of triply heterozygous loci that were imputed as homozygous. Increases precision
		cor = reImpute	Imputes missing phases if the homologous loci were imputed. Increases recall. Not recommended, use correctAll instead
		cor = correctAll	Combination of correctFalseHom and reImpute
Base pair threshold (thr)	Classifies recombination events as crossovers (>thr) or gene conversions (<thr) when flanking haploblock lengths lie under or over thr, respectively. Any thr ≥ 0	thr = 0	No discrimination applied. All recombination events classified as crossovers

**Fig. 1.**
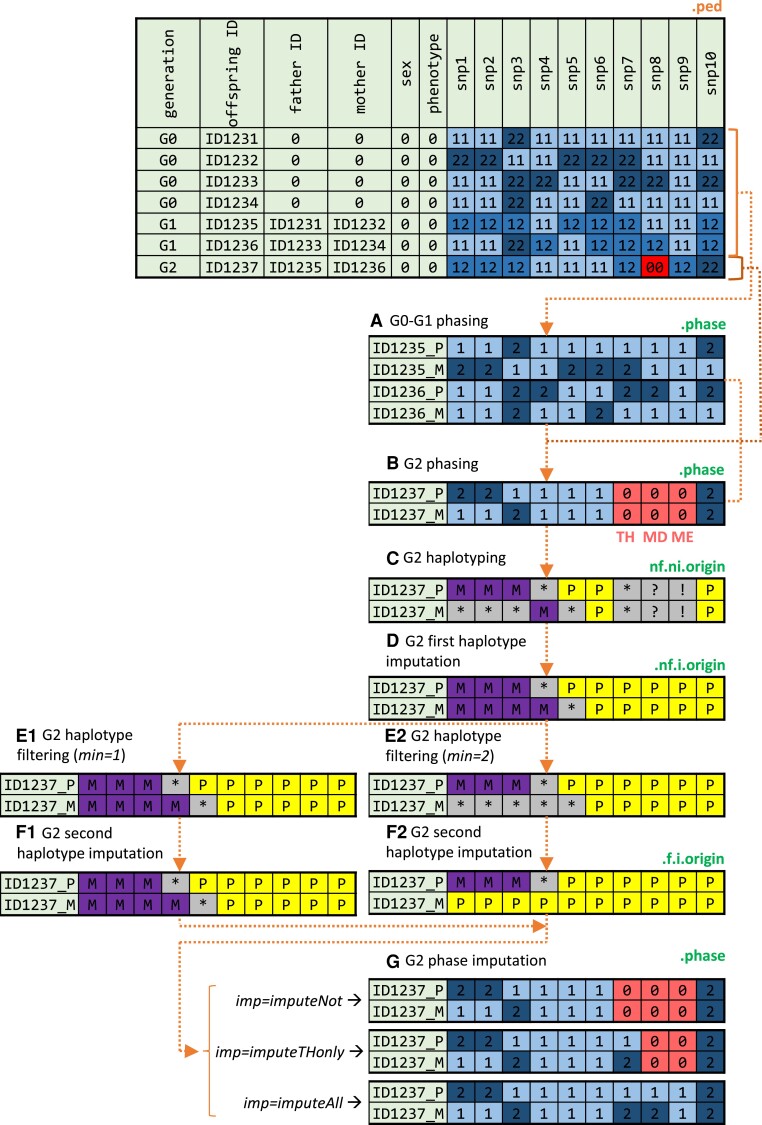
Example of the haploMAGIC workflow using an example dataset. The tables represent the input PED file (orange labels) and the output files of the a–g steps (green labels). The PED file contains the pedigree information (columns 1–4) and the diploid genotypes of 10 SNPs (columns 7–16) from the 8 members of a 3-generation (G0, G1, G2) family, where 0, 1, and 2 indicate missing, major, and minor allele, respectively. Each row of the output tables relates to a unique parental sequence (P, paternal; M, maternal) of the phase (indicated with the allele codes described before) or haplotype (P, paternal; M, maternal; *noninformative allele; ?, missing data; !, ME) of the G1 and G2 individuals. Alleles that could not be phased are colored red and labeled according to the phasing scenario (TH, triple heterozygote; MD, missing data; ME, Mendelian error). Noninformative alleles, where haplotype origins could not be assigned, are colored gray. Alternative paths depending on the options selected for the haploMAGIC arguments minimum number of informative allele, min, and phase imputation, imp (italic), are shown.

The haploMAGIC pipeline works iteratively from the generation of the founders, G0, until the last generation. In G0, when the phase of the founder lines is known due to homozygosity, the complete phase of the G1 offspring can be inferred directly (Fig. 1a). The G1 phases are compared with the unphased SNP genotypes of the G2 offspring by family trios using Mendelian segregation rules to reconstruct the phase (sequence of alleles sharing parental origin [Fig. 1b]). Phasing is not possible when (i) all trio members are heterozygous, (ii) one of the genotypes is missing, or (iii) due to Mendelian error (ME).

During the subsequent haplotyping step (Fig. 1c), haploMAGIC compares the phases of each offspring with the phase of the corresponding parents by trios to assign the grandparental haplotype origins of each allele. The origin of both unphased alleles and phased alleles descending from homozygous parents cannot be assigned. Therefore, we will refer to these alleles as noninformative. Next, haploMAGIC completes haploblocks by imputing the origin of noninformative alleles between alleles assigned with the same origin or in the border (Fig. 1d), filters haploblocks having less than the minimum number of informative alleles, min (Fig. 1e), and imputes the filtered origins again (Fig. 1f), retaining the most likely grandparental origin of each allele based on the pedigree and genotyping information available and on the selected threshold of the minimum number of informative alleles per haploblock (min). Optionally, unresolved phases can be imputed with those from the corresponding origins depending on the option selected of phase imputation, imp (Fig. 1g). The phase of triply heterozygous loci that were imputed incorrectly can be corrected and/or reimputed depending on the option selected of phase correction (cor).

haploMAGIC detects recombination events as transitions between grandparental origins, that is the regions between paternal and maternal haploblocks or vice versa, and records the start and the end SNP coordinates of the recombination intervals. Additionally, haploMAGIC assigns the origin of each allele to the founder lines by using the grandparental haplotype origins and the pedigree relationships. If a base pair threshold is provided, recombination events can be discriminated between COs and gene conversions.

### Simulations

#### Simulation parameters

An extension of AlphaSimR version 1.3.1 (Gaynor *et al*. 2021) designated simulate_chromosome.r was used to generate datasets of chromosomes with/or without GE rates to allow for the testing and comparison of haploMAGIC with duoHMM and LINKPHASE3. Ten GE rates (%) were tested (0, 0.5, 1, 2, 3, 4, 5, 6, 10, 15) in simulated chromosomes. Simulated datasets utilized the pedigree structure of each of the 2 available *Brassica napus* MPPs, populations 1 and 2, and the genomic position of the SNP markers on chromosomes A03, A04, and C09 (see Supplementary Table 1), resulting in a total of 60 simulations. Genetic distance was not utilized to allow comparison between software in equal conditions. The chromosomes were selected based on their diverse properties in terms of chromosome length, number of SNP loci, and mean SNP interval distance. Information about the MPPs and the SNP data is described in “*Real-world data*.”

The arguments selected for the Markovian Coalescent Simulator, run by the function runMacs(), were “species = GENERIC” to simulate a generic recombination model, SegSites equal to the number of loci in the input MAP file, and nInd as the number of founders in the input population. After this, the functions newPop() and pedigreeCross() generated simulated populations. GE rates were added with the function addErrors(). Errors were simulated in generations G2, G3, and G4. The simulated recombination events were considered as the truth in the performance analysis.

#### Software execution

For each simulation, haploMAGIC performance was analyzed for the corresponding combination of population, chromosome, minimum number of informative alleles per haploblock (1, 2, 3, 4), phase imputation option (imputeNot, imputeTHonly, imputeAll), and postimputation phase correction (correctAll, correctFalseHom). Note that imp = imputeNot forces cor = correctNot.

For the execution of duoHMM (version 0.1.7), the text PED file was converted using PLINK (version 1.90) to a binary BED file and then utilized as input of SHAPEIT (version 2.r904) (Delaneau *et al*. 2013) with arguments –duohmm, which applies a post hoc haplotype correction based on the pedigree structure, to produce a SHAPEIT graph. The Markov chain Monte Carlo arguments selected were –thread 20 –burn 10 –states 5 –prune 10 –main 50 –window 5. Next, we simulated 10 haplotype sets from the graph and used them to detect CO recombinations with duoHMM (O’Connell *et al*. 2014). CO estimations obtained with duoHMM were filtered with thresholds of probability of recombination that reduced the number of COs in a similar proportion to each haploMAGIC min threshold with constant imp option, imp = imputeNot.

For LINKPHASE3, the physical distances in bp were converted to genetic distances in cM following the recommended conversion rate 1 Mb = 1 cM (Druet and Georges 2015). For the software execution, the default parameters from the user's manual were maintained, which are HALFSIB_PHASING = yes, applying linkage to reconstruct the parental haplotypes based on segregation of marker alleles in offsprings, HMM_PHASING = yes, to improve haplotype reconstruction in the presence of GEs, N_TEMPLATES = 50, for within-family imputation, and CHECK_PREPHASING = yes, to improve haploblock estimation. For the output analysis, we used the file “recombinations_hmm” instead of “recombinations” because of its better predictions, as reported previously (Druet and Georges 2015).

#### Quantification of performance with output from simulations

The precision, recall, *F*1 score, and adjusted resolution were calculated for each set of predicted recombination events and averaged by method and GE rate. Subsequently, we calculated the number of simulated COs that were detected, i.e. when the coordinate of a simulated CO was found within the start and the end coordinates of a detected recombination event. This number was divided either by the number of detected COs or by the number of simulated COs for the calculation of precision and recall, respectively. When 2 or more simulated COs were associated with the same detected event, then all of them were counted as simulated but only 1 as simulated and detected. *F*1 scores were calculated with the formula:


$$F1\;\mathrm{score}=\;\frac{2\times \mathrm{Precision}\times \mathrm{Recall}}{\mathrm{Precision}+\mathrm{Recall}}$$

Differences in precision between methods and/or error rates were adjusted by multiplying resolution with precision. Resolution is defined as the inverse of the median number of loci per recombination interval. Adjusted resolution can be explained as the probability of an SNP interval within the interval of a detected recombination event to be the locus of this recombination event in case the detected event is true.


$$\mathrm{Adjusted}\;\mathrm{resolution}=\;\mathrm{Precision}\times \mathrm{Resolution}=\;\frac{\mathrm{Precision}}{\begin{array}{c} \mathrm{Median}\;\mathrm{num}.\;\mathrm{loci}\;\mathrm{per}\;\\ \mathrm{recombination}\;\mathrm{interval} \end{array}}$$

The percentage of recombination events that were removed by filtering compared with the raw output for haploMAGIC was calculated after every run and used to select thresholds of probability of recombination of duoHMM that produce similar outcome.

#### Real-world data

Both haploMAGIC and LINKPHASE3 were applied on the SNP data of 2 large MPPs of *B. napus*, as they both showed good performance with MPPs in the simulation. The populations 1 and 2 had 1,327 and 1,413 individuals, respectively. The populations were developed from homozygous founder lines (generation G0) following a chain-crossing scheme until the fifth generation (G4) and full pedigree information was available (Krenzer *et al*. 2024) (see Supplementary Fig. 1). Generations G0, G2, G3, and G4 were genotyped using a 15 K SNP array (Clarke *et al*. 2016) (including 13,714 SNPs) with markers targeting loci on the 19 chromosomes of the *B. napus* genome (AACC, 2*n* = 4*x* = 38). The genotypes of the G1 hybrids were inferred from the inbred founder parents.

SNP flanking sequences (Scheben *et al*. 2019) were aligned to the *B. napus* reference genome *Express 617* v1 (Scheben *et al*. 2019; Lee *et al*. 2020). SNPs were further filtered to eliminate markers with >10% ME rate, resulting in 11,443 SNPs. ME rates were measured within each population and by generations. In order to select the best haploMAGIC setting, ME rates calculated in populations were converted to GE rates following the linear relationship between both rates (Hao *et al*. 2004) by calibrating to reference curves obtained with the AlphaSimR simulations with different ranges of GE rates (Saunders *et al*. 2007).

For haploMAGIC, the effect of different phase imputation methods on the output was tested by comparing 2 combinations of phase imputation and phase correction, being imp = imputeTHonly/cor = correctFalseHom and imp = imputeAll/cor = correctAll. For the minimum number of informative alleles per haploblock, different options were applied, namely min = 2, min = 3, and an alternative setting consisting of adjusted thresholds for each generation based on their specific GE rates, min = 2/5/3, i.e. min thresholds 2 for G2, 5 for G3, and 3 and G4. The alternative approach aims to enhance the consistency of CO numbers per gamete across generations that would otherwise show inconsistencies due to varying ME rates. For the output of both programs, different parameters were calculated, such as the number of COs per gamete, both as the median value per generation and the global median, the mean percentage of informative alleles within duos per generation, and the percentage of filtered recombination events, as the mean value per generation as well as the global mean.

## Results and discussion

### Performance comparison between haploMAGIC and duoHMM with simulated data

**Fig. 2.**
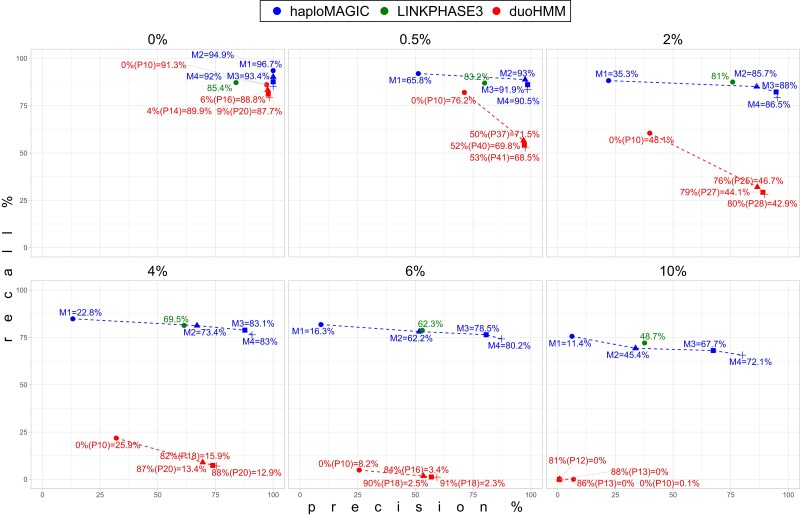
Comparison of the precision and recall scores of haploMAGIC, LINKPHASE3, and duoHMM. Each graph shows the performance with 6 (0, 0.5, 2, 4, 6, 10%) out of the 10 simulated genotyping error rates. haploMAGIC labels show “M” followed by the minimum number of informative alleles per haploblock (min) threshold and *F*1 score separated by equal sign. The haploMAGIC values shown here were obtained with the imp = imputeNot and cor = correctNot. Labels represent *F*1 score values in the LINKPHASE3 points. For the duoHMM points, labels show the percentage of filtered recombination events whose probability passed the threshold percentage of recombination probability, in parentheses after “P”, and the *F*1 score after the equal sign. Points of haploMAGIC had a comparable percentage of recombination events filtered out as the duoHMM point they share shape with.

haploMAGIC consistently outperformed duoHMM with thresholds min = 2, min = 3, and min = 4 for the minimum number of informative alleles per haploblock (Fig. 2; see Supplementary Tables 2, 3, 5, and 6). haploMAGIC only obtained lower *F*1 scores when no filtering was applied (min = 1) with the GE rates 0.5–4%. Filtering increased the precision of CO detection considerably for both haploMAGIC and duoHMM. However, this increase in precision was associated with a larger recall drop of duoHMM, compared with haploMAGIC. Increasing filtering stringency does not translate to higher *F*1 scores for duoHMM as opposed to haploMAGIC. The lower precision could be due to duoHMM not utilizing pedigree information for phasing, but for correcting phasing errors after SHAPEIT2 assuming unrelatedness.

### Performance comparison between haploMAGIC and LINKPHASE3 with simulated data

haploMAGIC outperformed LINKPHASE3 with thresholds min = 3 and min = 4 for the minimum number of informative alleles per haploblock (Fig. 2; see Supplementary Tables 2–4). LINKPHASE3 obtained higher *F*1 scores under any GE rate without filtering (min = 1) and with GE rates up to 6% when haploblocks with <2 informative alleles were removed (min = 2). However, haploMAGIC obtained consistently higher adjusted resolution relative to LINKPHASE3 (see Supplementary Tables 2–4).

Compared with duoHMM, LINKPHASE3 performed consistently better in the presence of GEs. This might be a result of LINKPHASE3 utilizing the pedigree information and applying Mendelian segregation rules directly for phasing and CO detection, making it less prone to switch error and facilitating GE detection. Nevertheless, the implementation of a hidden Markov model by LINKPHASE3 is less stringent discarding false-positive haploblocks caused by GEs. Furthermore, LINKPHASE3 lacks options to discard COs with low confidence, as opposed to the information on the probability of recombination provided by duoHMM, which appears to limit the maximum precision that can be achieved. To address this limitation, haploMAGIC provides different levels of filtering to accommodate performance despite varying GE rates.

### haploMAGIC performance with real-world data

GE rates of ∼3.5 and 3.0% were estimated for populations 1 and 2, respectively. However, the distribution was not homogeneous across generations but instead notably higher in G3 (Table 2). Adjusting the required number of informative alleles to keep haploblocks by generations based on these generation-specific ME rates produced the lowest difference between generations in terms of CO number per gamete as well as the global standard deviation (min = 2/5/3 for G2/3/4, respectively). These results appeared to have overcome the artifacts likely caused by GEs. These errors could not be eliminated with fixed filtering threshold across generations.

**Table 2.** Median CO number per gamete obtained with haploMAGIC or LINKPHASE3 and ME rates (%) in each real-world population (1, 2) and generation (G2, G3, G4).

Population	Generation	haploMAGIC						LINKPHASE3	ME rate (%)
		min = 2		min = 3		min = 2/5/3			
		imputeAll	imputeTHonly	imputeAll	imputeTHonly	imputeAll	imputeTHonly		
1	G2	24.0	24.0	19.0	19.0	24.0	24.0	22.5	1.24
2	G2	26.0	26.0	21.0	21.0	26.0	26.0	20.0	0.87
1	G3	109	94.0	75.5	65.0	47.0	40.0	67.0	8.54
2	G3	109	95.0	75.5	66.0	46.0	40.0	63.0	8.81
1	G4	67.0	33.0	49.0	24.0	50.0	24.0	41.0	1.21
2	G4	69.0	36.0	51.0	27.0	51.0	27.0	41.0	0.95
Median		68.0	39.0	50.0	29.0	43.0	30.0	42.0	
SD		34.3	30.8	22.7	20.3	11.2	7.03	17.9	

Values are shown for different haploMAGIC settings of the methods minimum number of informative alleles per haploblock to be retained (min) and phase imputation (imp). The min thresholds shown are min = 2, min = 3 and min = 2/5/3, a combination of 3 different min thresholds for each generation. The imp options imp = imputeAll and imp = imputeTHonly were followed by the postimputation phase correction (cor) options cor = correctAll and cor = correctFalseHom, respectively, and LINKPHASE3. The last 2 rows show the global median and standard deviation values.

haploMAGIC output was influenced by the selected filtering method. Imputing all unresolved phases from the context origins (imp = imputeAll) increased the per generation median and standard deviation of the number of COs (Table 2). Imputing the phase of only triply heterozygous loci (imp = imputeTHonly) produced more comparable CO number median values between the generations with lower GE rates, G2 and G4, than when all unresolved phases were imputed (imp = imputeAll). Given the higher error rates in G3, imputing the offsprings’ phase from the parental phases might generate false-positive COs in G4 when the errors were due to false parental genotypes. Not imputing (imp = imputeNot) or partially imputing unresolved phases (imp = imputeTHonly) results in the greater increase in the proportion of noninformative alleles (Table 3) and can therefore improve precision under challenging GE rates (See Supplementary Tables 2 and 3).

**Table 3.** Percentage of noninformative alleles per meiosis and percentage of recombination events that were filtered based on the minimum number of informative alleles per haploblock to be retained (min) (parenthesis) obtained in each real-world population (1, 2) and generation (G2, G3, G4) with different haploMAGIC settings of the methods minimum number of informative alleles per haploblock to be retained (min) and phase imputation (imp).

Population	Generation	min = 2		min = 3		min = 2/5/3	
		imputeAll	imputeTHonly	imputeAll	imputeTHonly	imputeAll	imputeTHonly
1	G2	79.9 (55.1)	79.9 (55.1)	79.9 (67.0)	79.9 (67.0)	79.9 (55.1)	79.9 (55.1)
2	G2	80.2 (53.6)	80.2 (53.6)	80.2 (63.4)	80.2 (63.4)	80.2 (53.6)	80.2 (53.6)
1	G3	81.5 (47.2)	83.8 (46.4)	81.4 (63.2)	83.7 (62.6)	81.5 (77.2)	83.8 (77.1)
2	G3	81.5 (46.3)	83.6 (45.8)	81.5 (62.5)	83.5 (62.1)	81.5 (77.0)	83.6 (76.8)
1	G4	86.6 (44.4)	92.1 (42.0)	86.3 (60.2)	91.7 (58.7)	86.4 (60.9)	92.0 (59.0)
2	G4	86.8 (44.1)	91.8 (41.5)	86.5 (60.1)	91.5 (56.7)	86.2 (60.7)	91.7 (57.0)

The min thresholds shown are 2, 3, and 2/5/3, a combination of 3 different min thresholds for each generation. The imp options imputeAll and imputeTHonly were followed by the postimputation phase correction (cor) options correctAll and correctFalseHom, respectively.

**Fig. 3.**
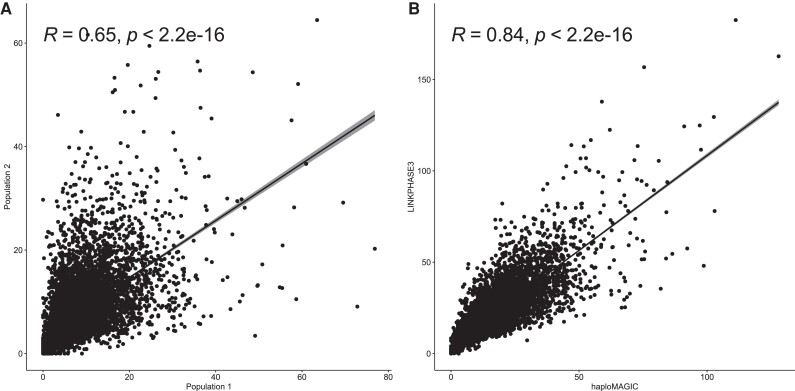
Spearman correlation between the genome-wide number of COs detected in (a) the real-world populations 1 and 2, detected with haploMAGIC (min = 2/5/3, imp = imputeTHonly, cor = correctFalseHom) and in (b) the real-world populations 1 and 2 with haploMAGIC (min = 2/5/3, imp = imputeTHonly, cor = correctFalseHom) and with LINKPHASE3 (output from recombinations_hmm). The dots represent the number of recombination events detected per SNP interval in all meioses. When recombination gaps spanned over several SNP intervals, the number was normalized as the inverse of the number of SNP intervals within the same recombination gap, so that the CO number in each recombination gap summed to 1.

Combining phase imputation of only triply heterozygous loci (imp = imputeTHonly) with the adjusted filtering by generations (min = 2/5/3) yielded the second lowest median number of CO per gamete and the lowest standard deviation between generations (Table 2). This suggests a median 1.58 COs per chromosome per meiosis, comparable to the 1.51 (Sun *et al*. 2007), 0.7 (Bayer *et al*. 2015), and 1.2 (Yan *et al*. 2023) COs per meiosis per chromosome reported previously in *B. napus*. This haploMAGIC setting achieved a low CO number after filtering ∼77% of the COs in G3 (Table 2). The results obtained with these parameters in populations 1 and 2 exhibited high Spearman’s correlation coefficients and similar genome-wide patterns of CO numbers between the 2 populations (Fig. 3a; see Supplementary Fig. 2).

### Comparison between haploMAGIC and LINKPHASE3 using real-world data

The patterns in CO number obtained with LINKPHASE3, with uneven numbers between G2 and G4, are similar to the results obtained with haploMAGIC when all unresolved loci were imputed (imp = imputeAll), particularly with additional filtering of haploblocks with less than 3 informative alleles (min = 3) (Table 2). Furthermore, the Spearman correlation test between the haploMAGIC and LINKPHASE3 results showed high correlation coefficients (Fig. 3b; see Supplementary Fig. 3). This suggests that despite haploMAGIC discarding many of the false-positive COs predicted by LINKPHASE3, both tools share a substantial overlap among the detected COs.

### Conclusion

Our benchmarking analysis showed that haploMAGIC outperformed related software in the process of identifying recombination events in terms of *F*1 score and resolution. Furthermore, haploMAGIC was found to retain consistency even with increasing GE rates. These improvements can be attributed to critical steps in the haploMAGIC pipeline, particularly haploblock filtering and (to a lesser extent) phase imputation. These options could be adjusted according to the expected likelihood of false-positive COs, which is dependent on the GE rate. Additionally, haploMAGIC was able to track alleles back to the founders and differentiate recombination events between COs and gene conversions.

## Supplementary Material

jkae109_Supplementary_Data

## Data Availability

Code and example data are available at: https://github.com/GoliczGenomeLab/haploMAGIC. Everyone is permitted to copy and distribute verbatim copies of this license document, but changing it is not allowed. Supplemental material available at G3 online.
