# Supplementary material for: haploMAGIC: accurate phasing and detection of recombination in multiparental populations despite genotyping errors
Source: G3 (Bethesda). 2024 May 29;14(8):jkae109. doi: 10.1093/g3journal/jkae109 (PMC11304941; doi:10.1093/g3journal/jkae109)

Figure S1 Crossing scheme, which was used to develop the two real-world populations. Each individual of G0 was crossed with two other founder individuals, resulting in generation G1, using a crossing scheme designed to maximise the diversity of each pairwise crosses within each population. Likewise, each individual of G1 was then crossed with two other G1 individuals, resulting in generation G2 composed of unique 4-way crosses. Ten plants from each cross in G2 were genotyped with the 15K-SNP Illumina Infinium Brassica napus genotyping array by SGS TraitGenetics, Gatersleben, Germany. Based on maximum inter-cross diversity, only one G2 plant from each family was selected to generate the next generation. The G3 and G4 generations were produced by repeating the same crossing and selecting procedure. As for G2, ten plants from each of the G3 and G4 families in each of the two populations were also genotyped with the 15K SNP array.


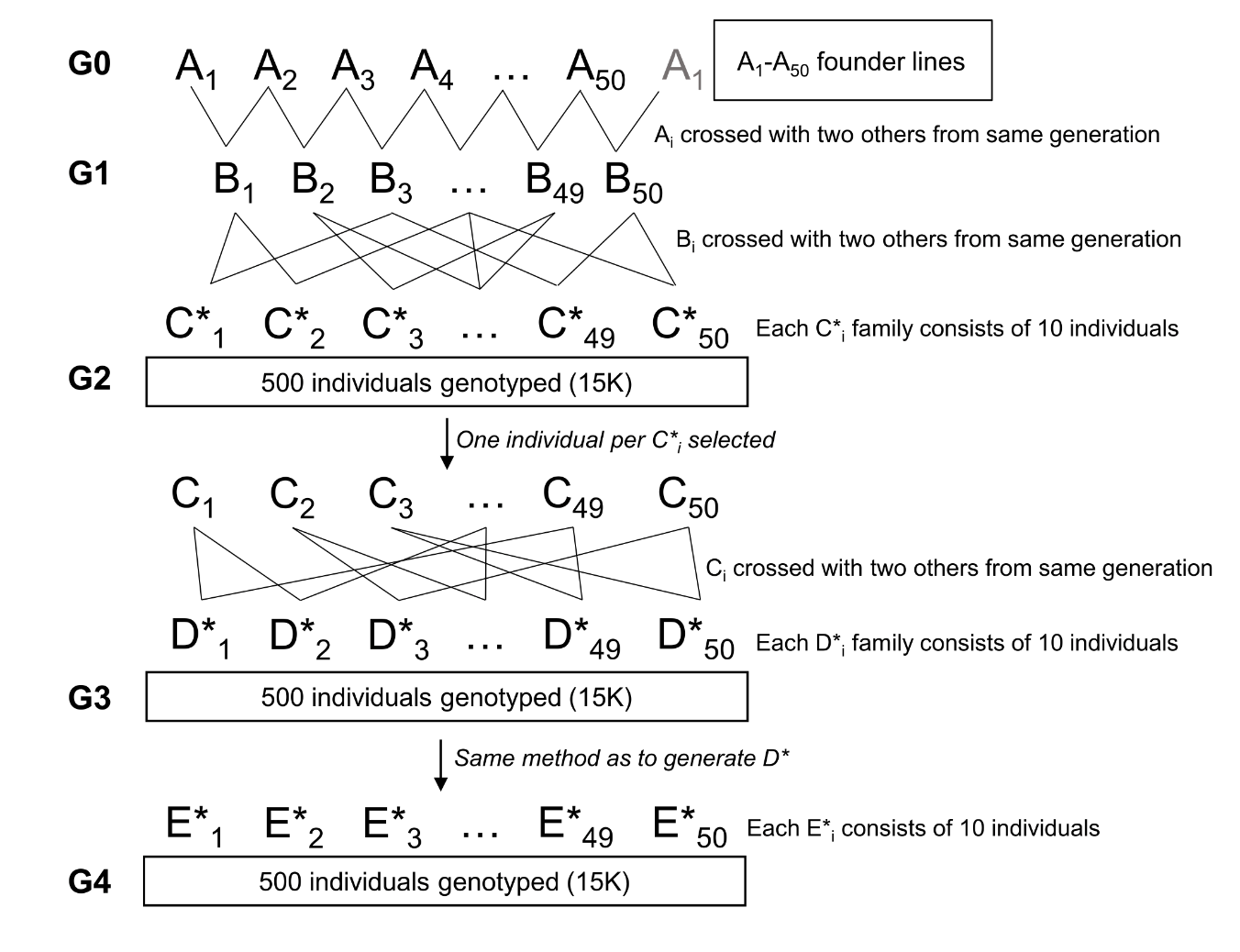


Figure S2 Distribution of the number of crossovers per SNP interval detected by haploMAGIC (min=2/5/3, imp=imputeTHonly, cor=correctFalseHom) in every informative meiosis in each of the two real-world populations, population 1 and population 2 across the 19 Brassica napus chromosomes, for which positions are indicated in base pairs. For each crossover gap, the measured numbers were normalized as the inverse of the number of SNP intervals within the gap, so that the crossover number in any recombination gap summed 1.


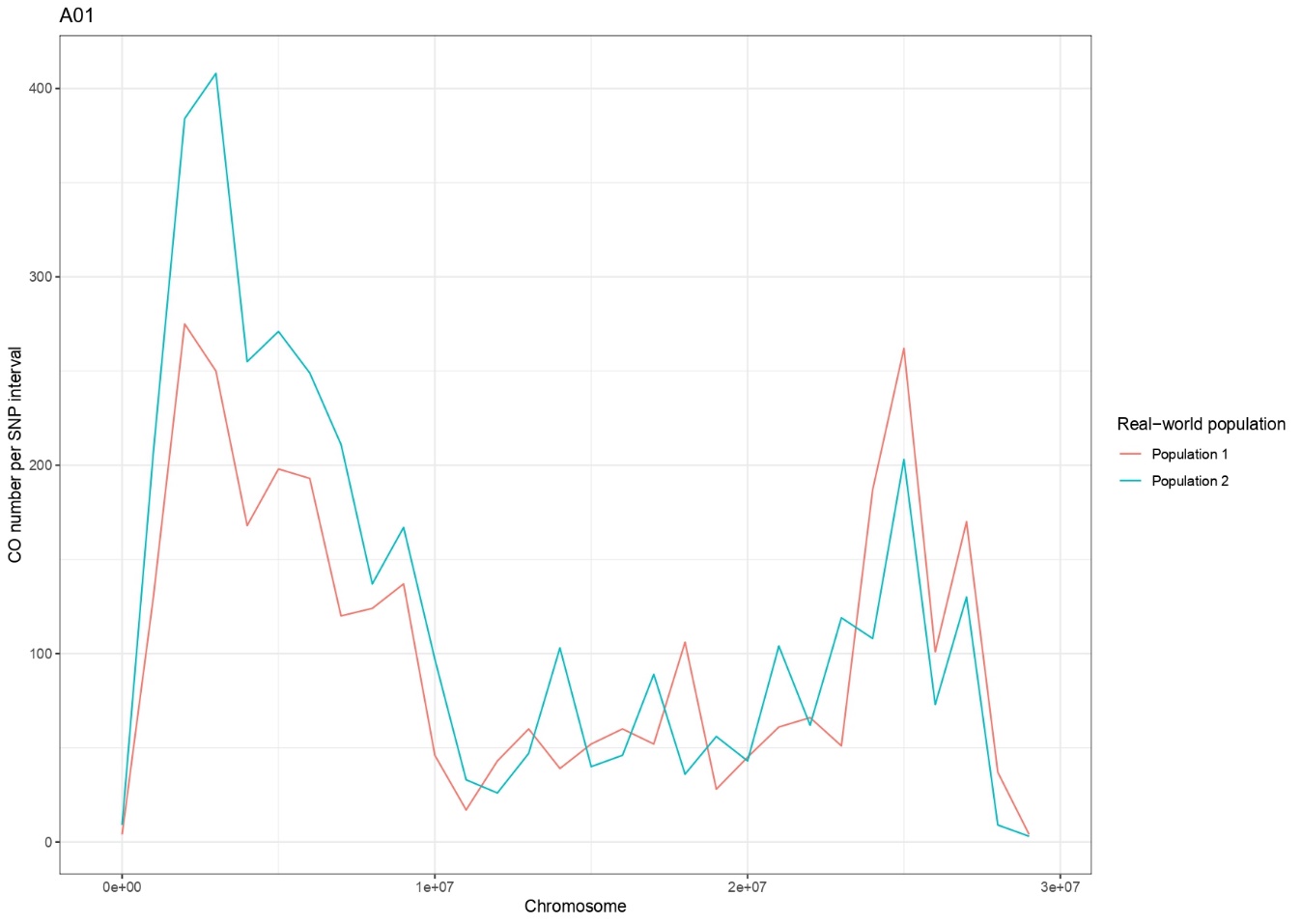


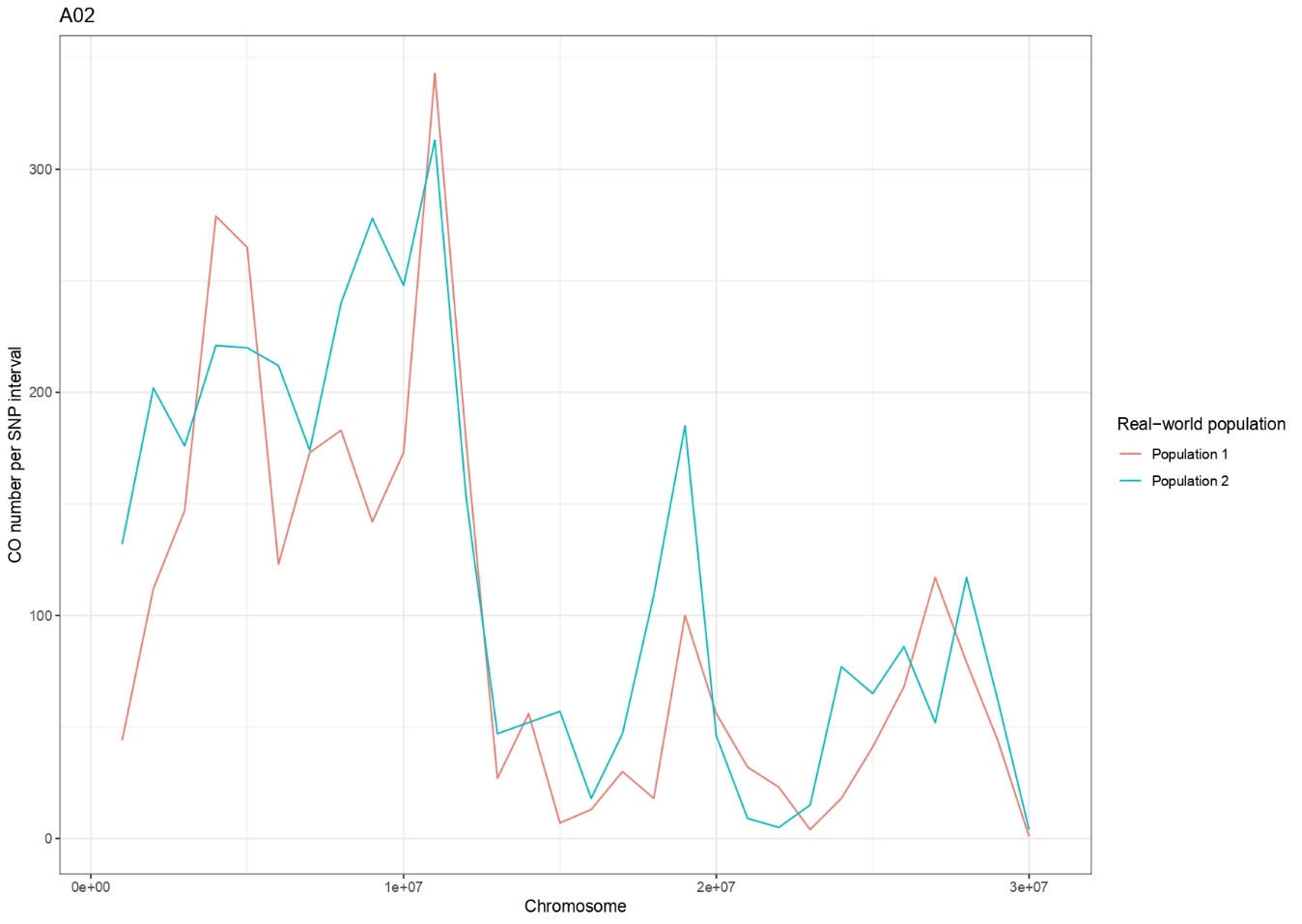

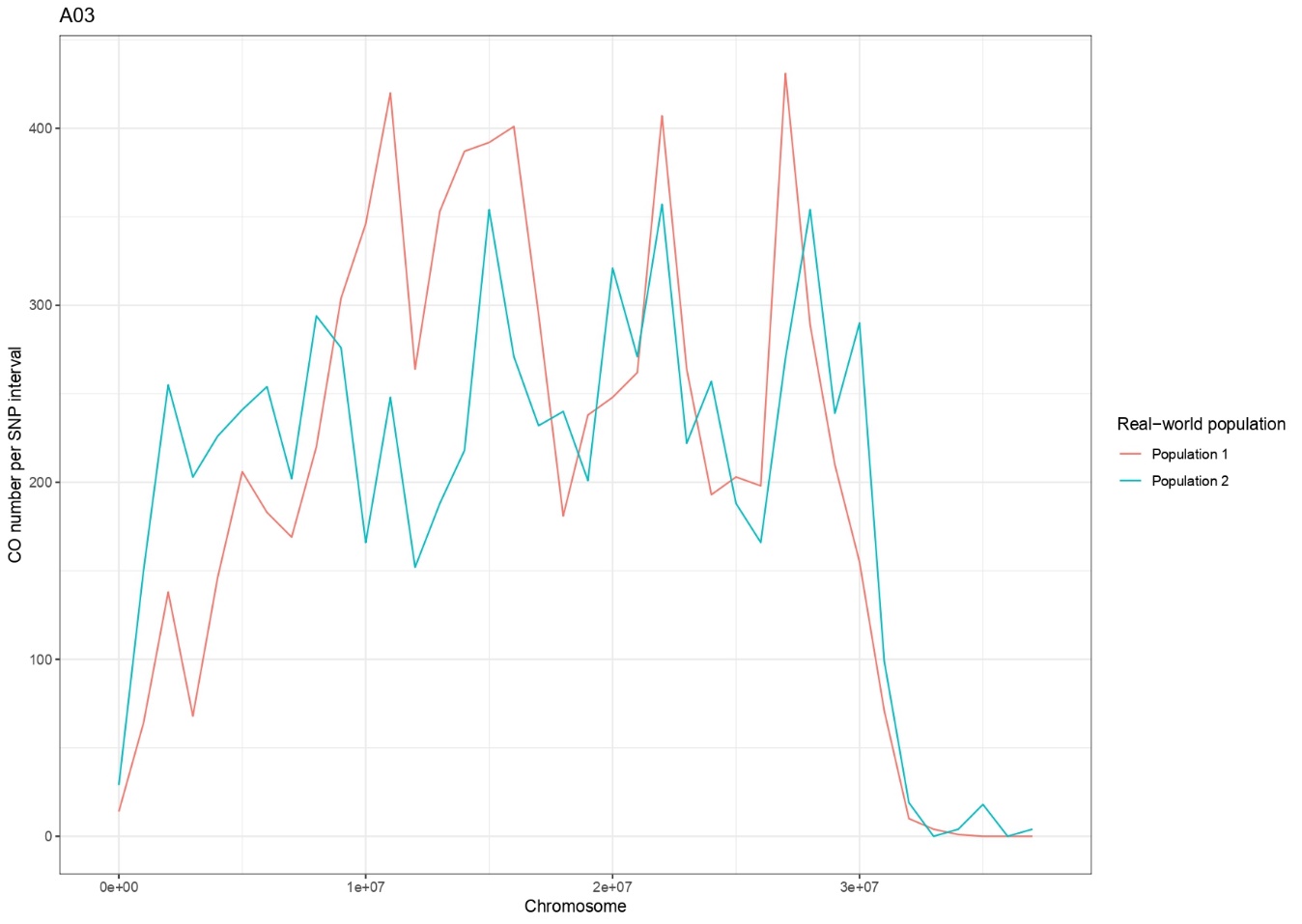

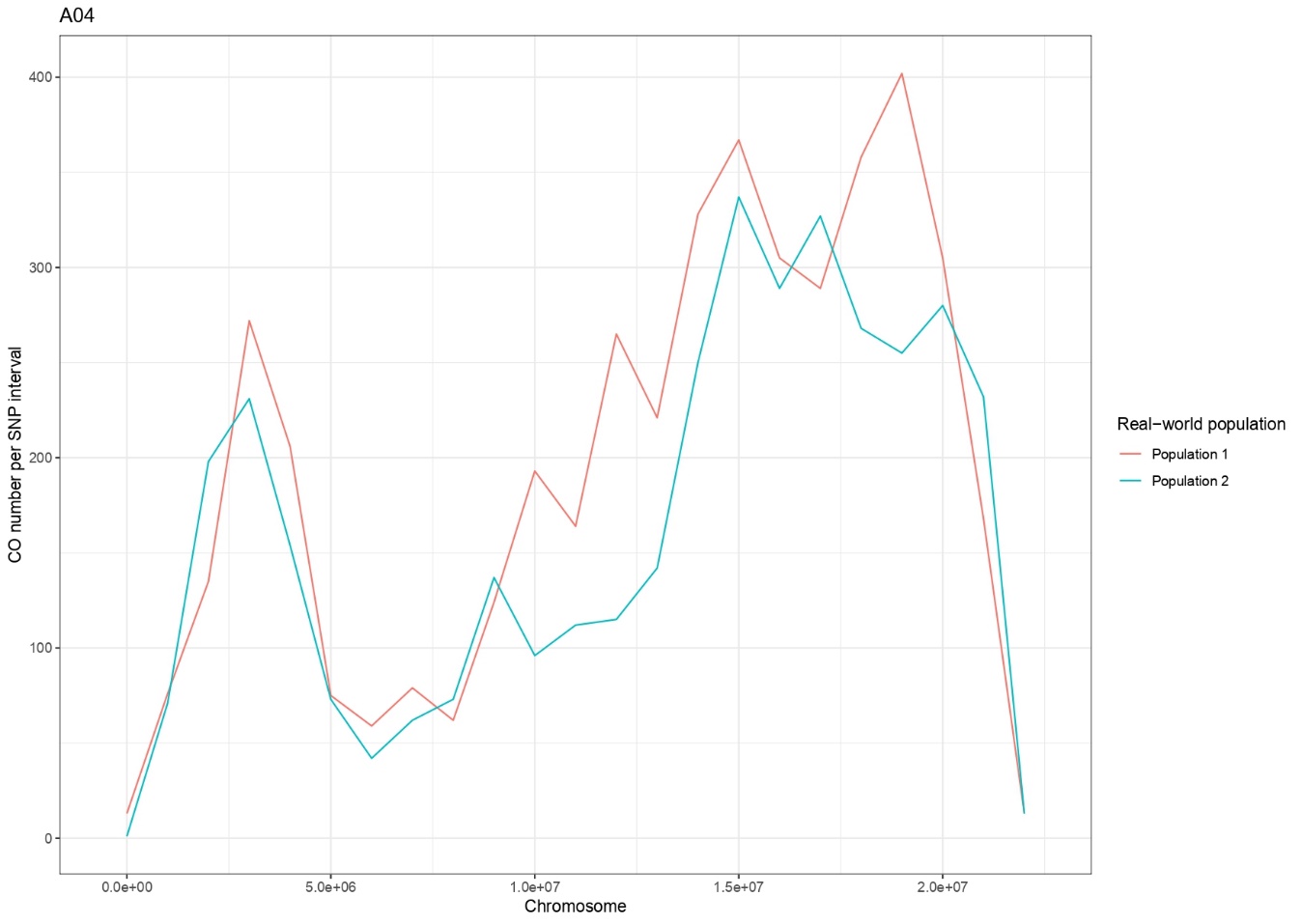

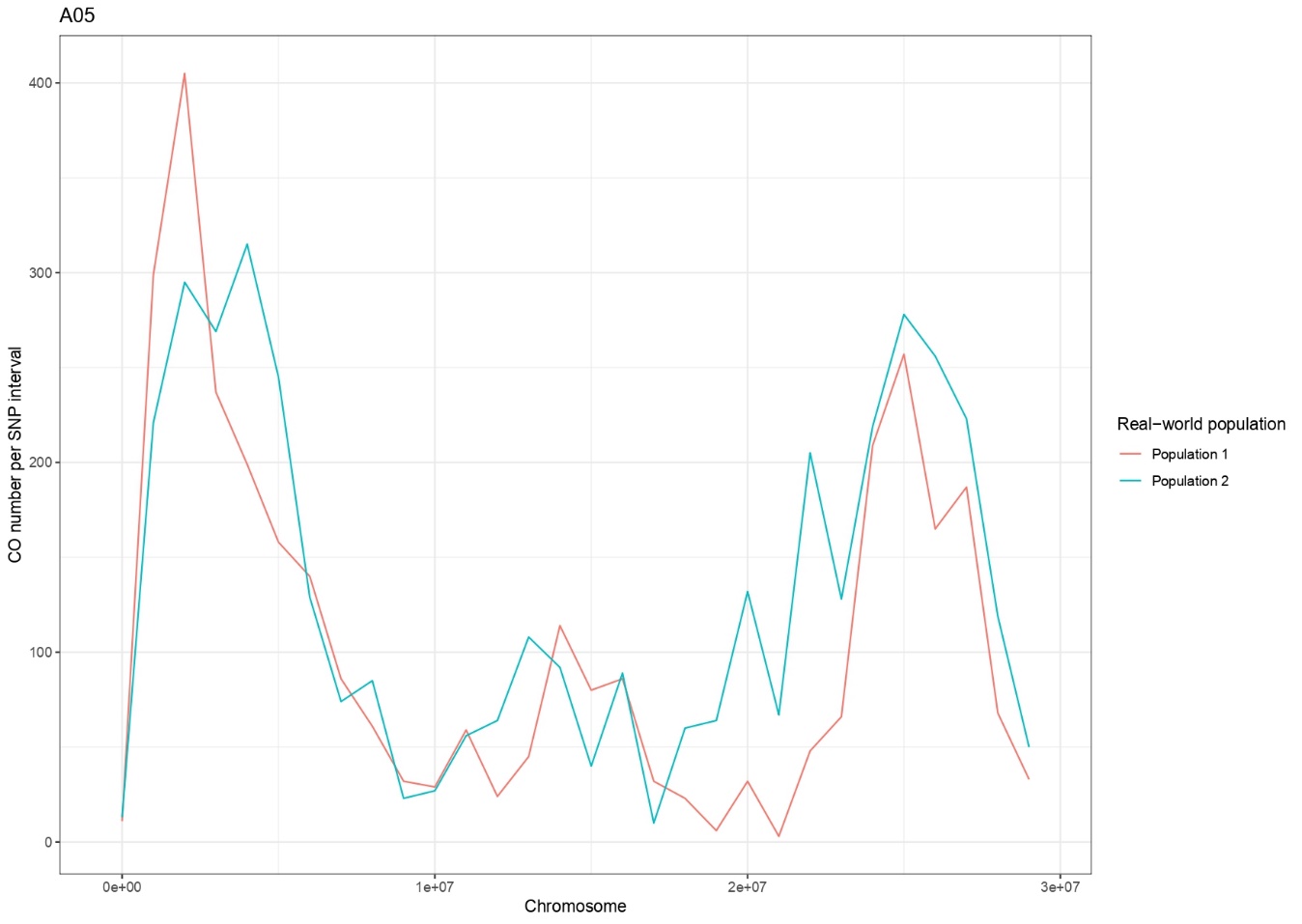

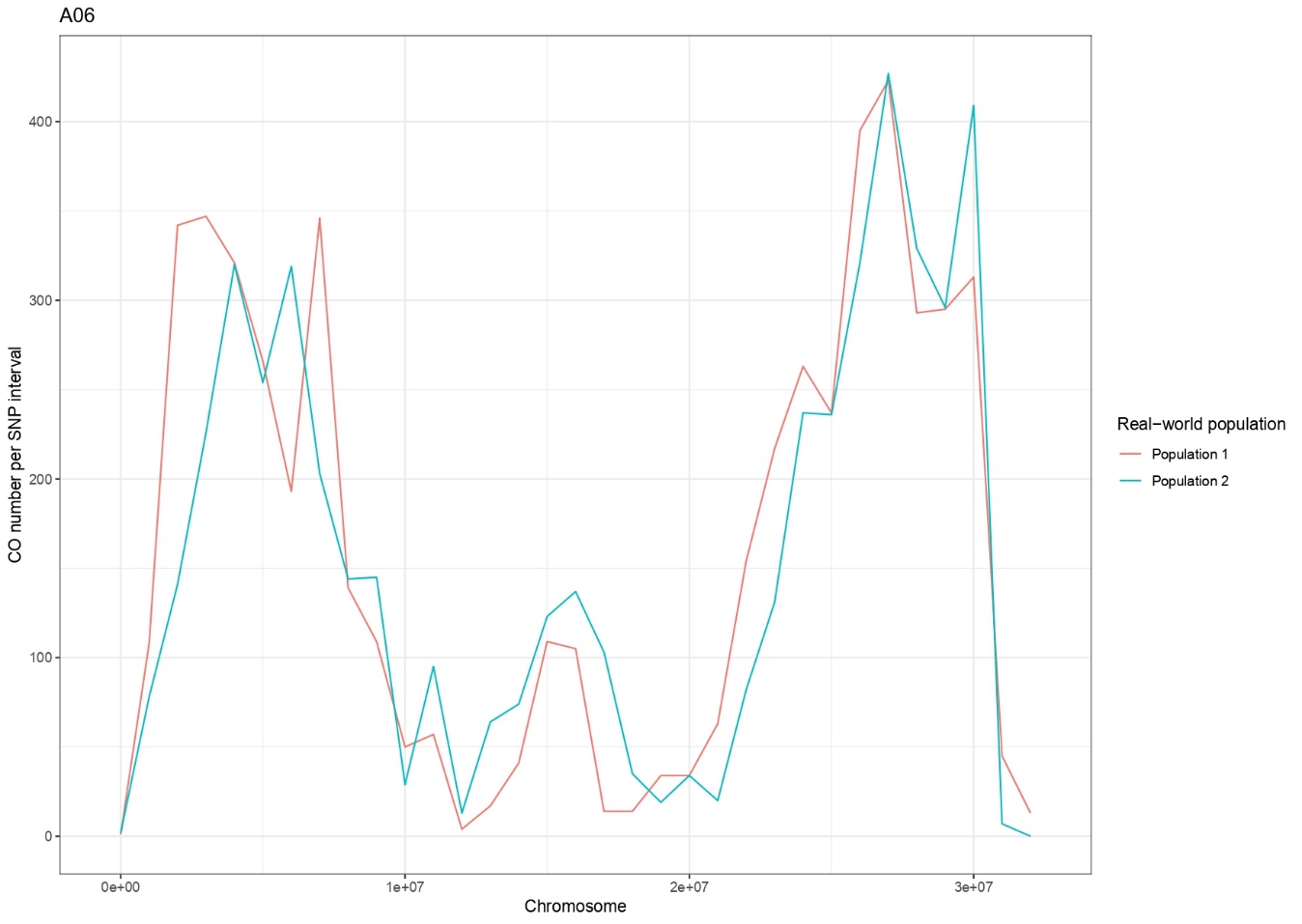

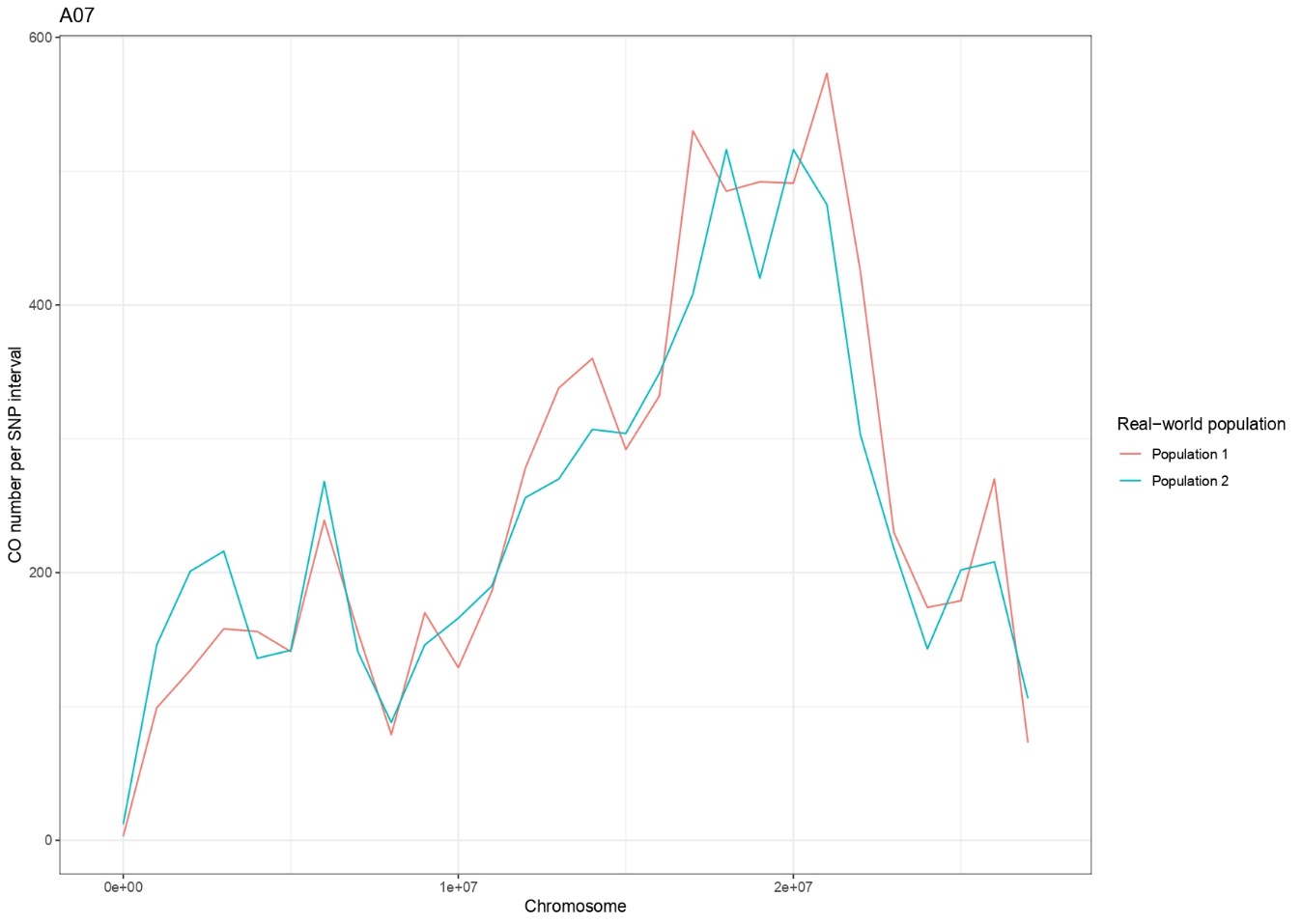

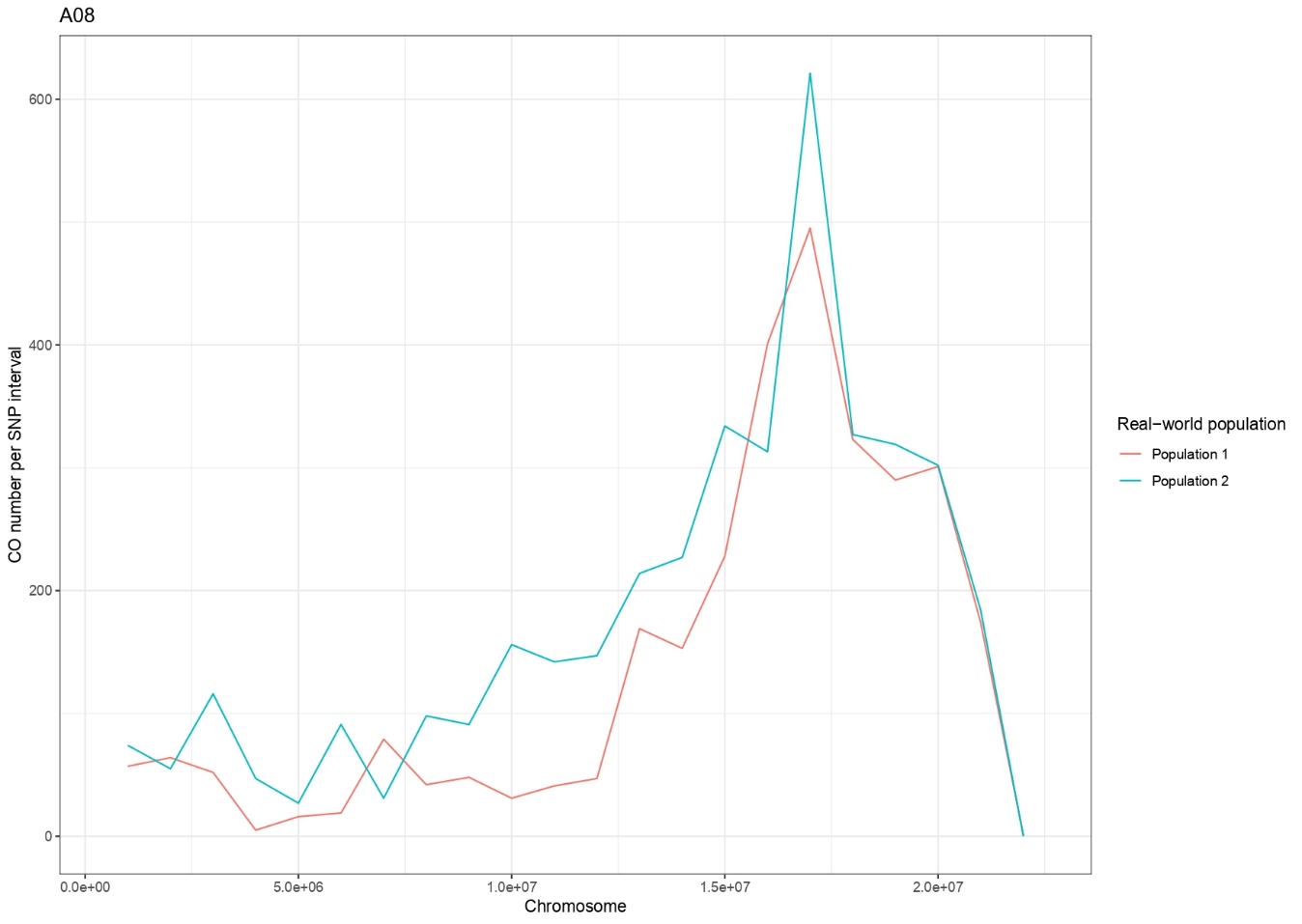

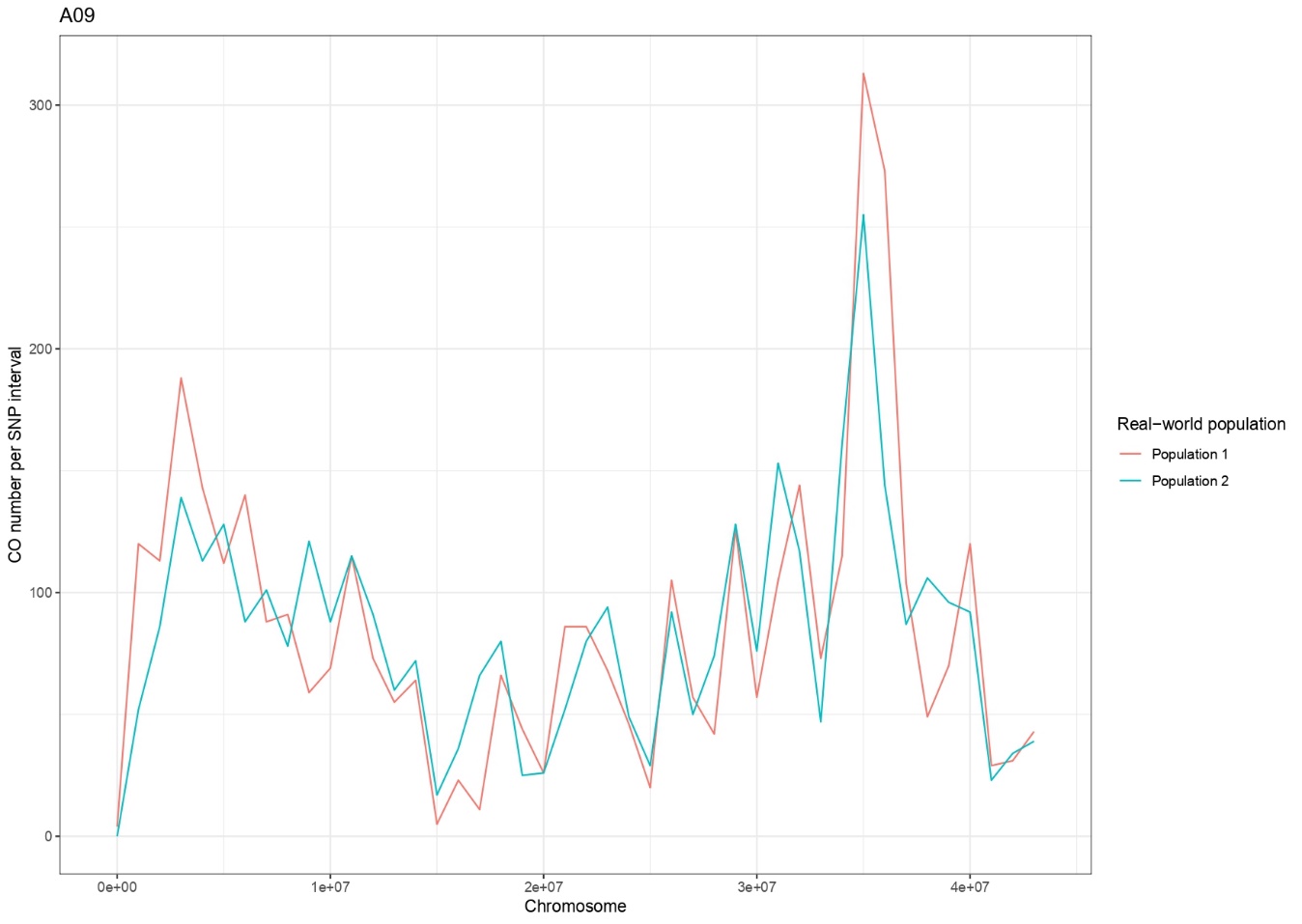

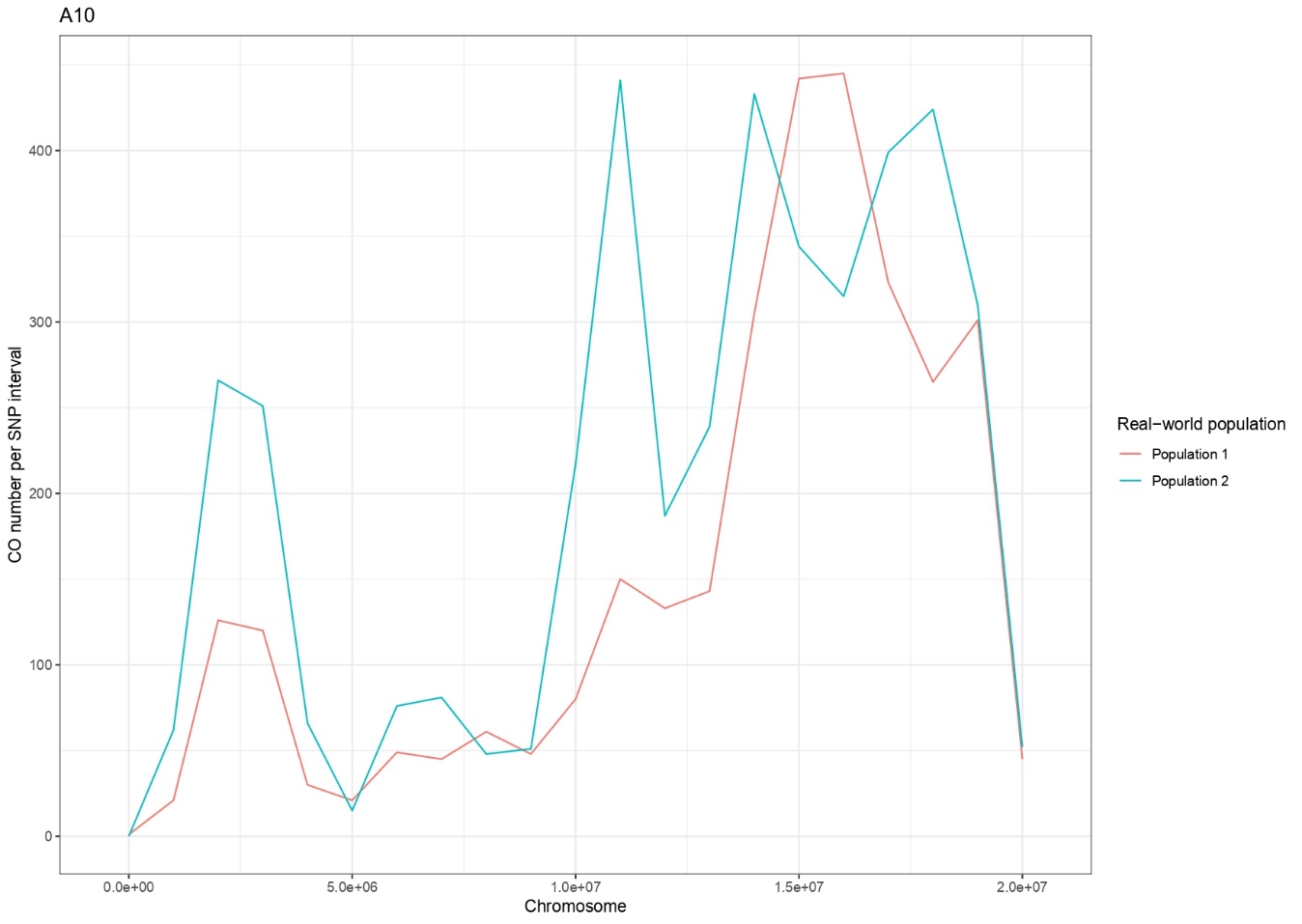

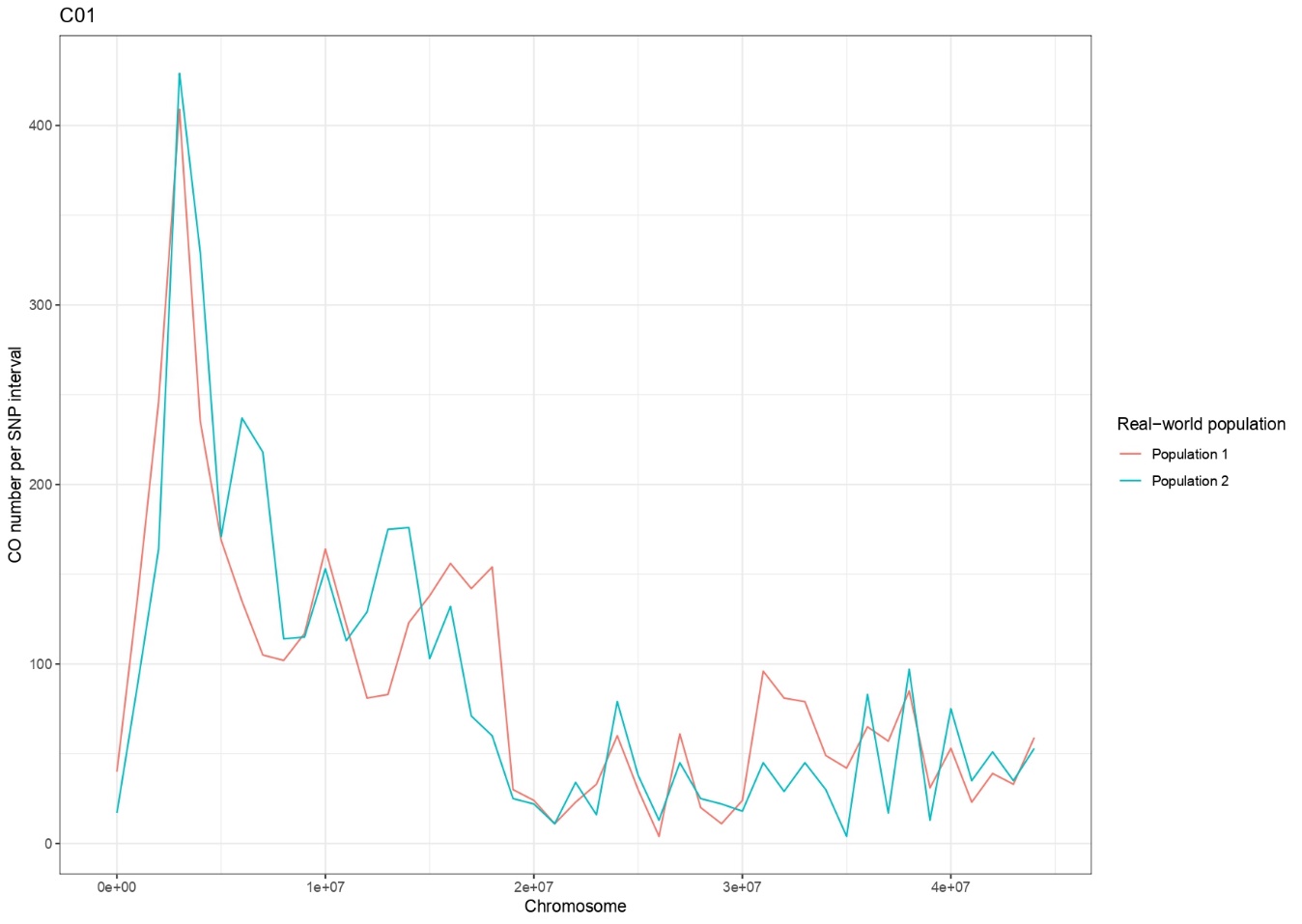

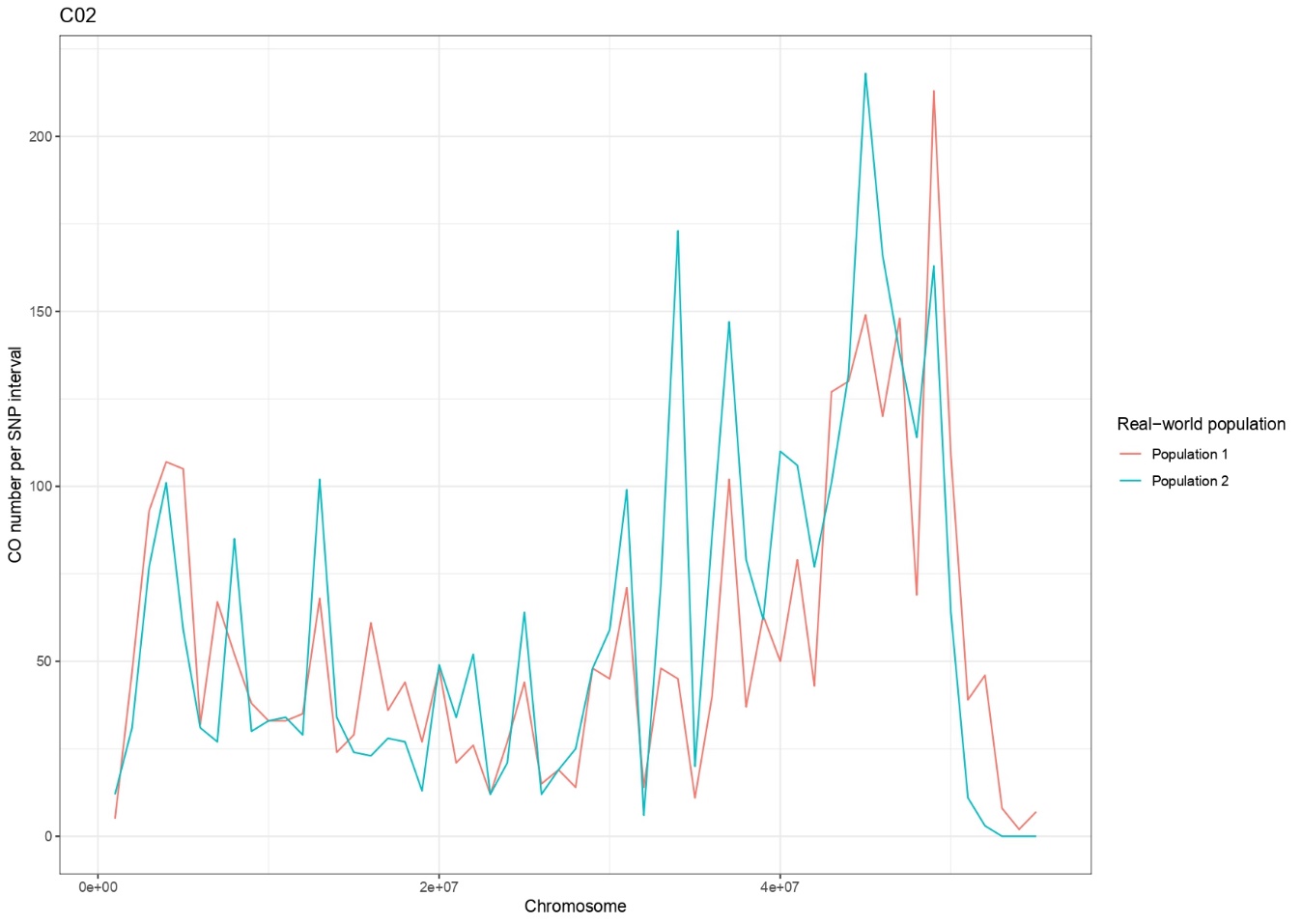

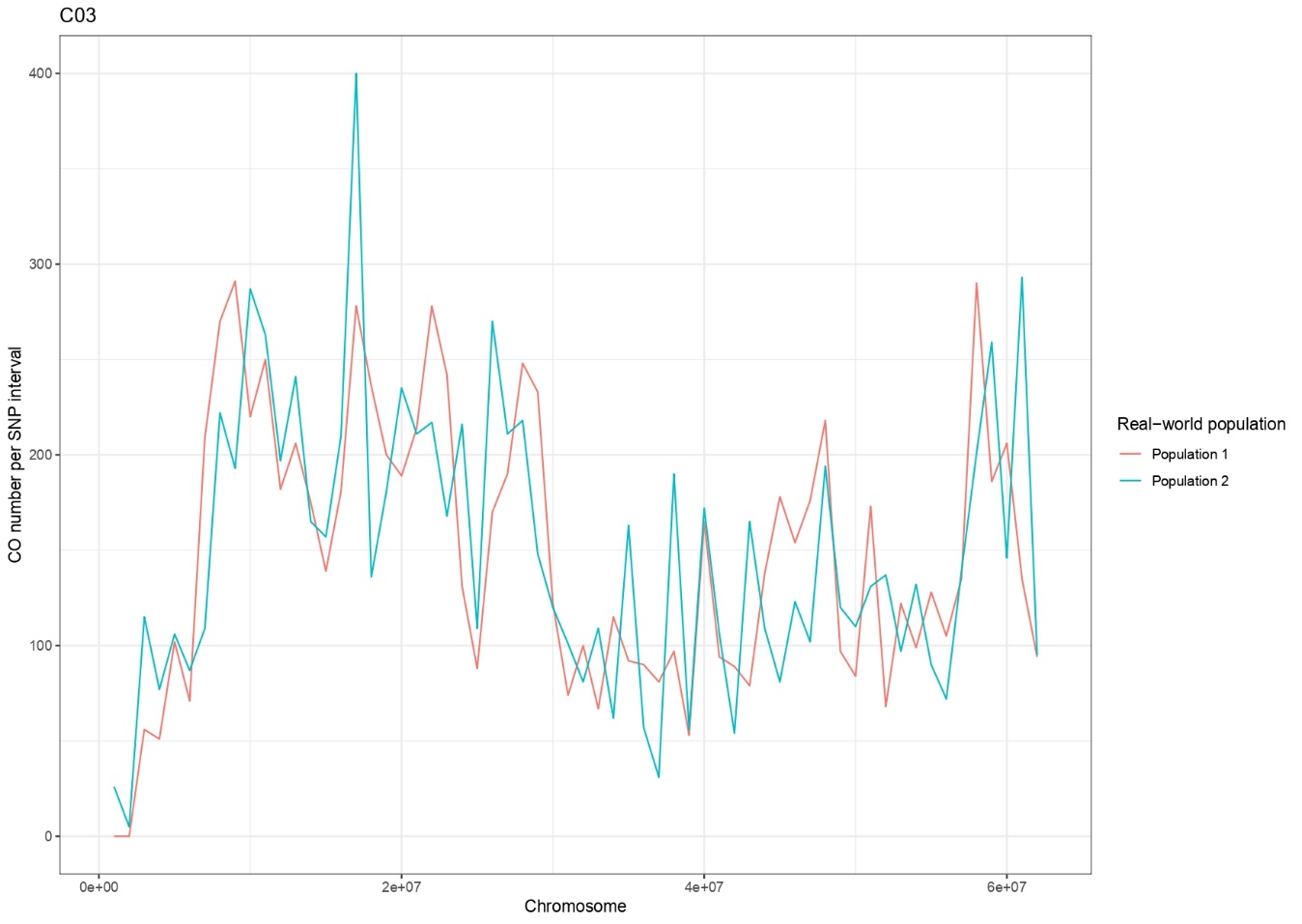

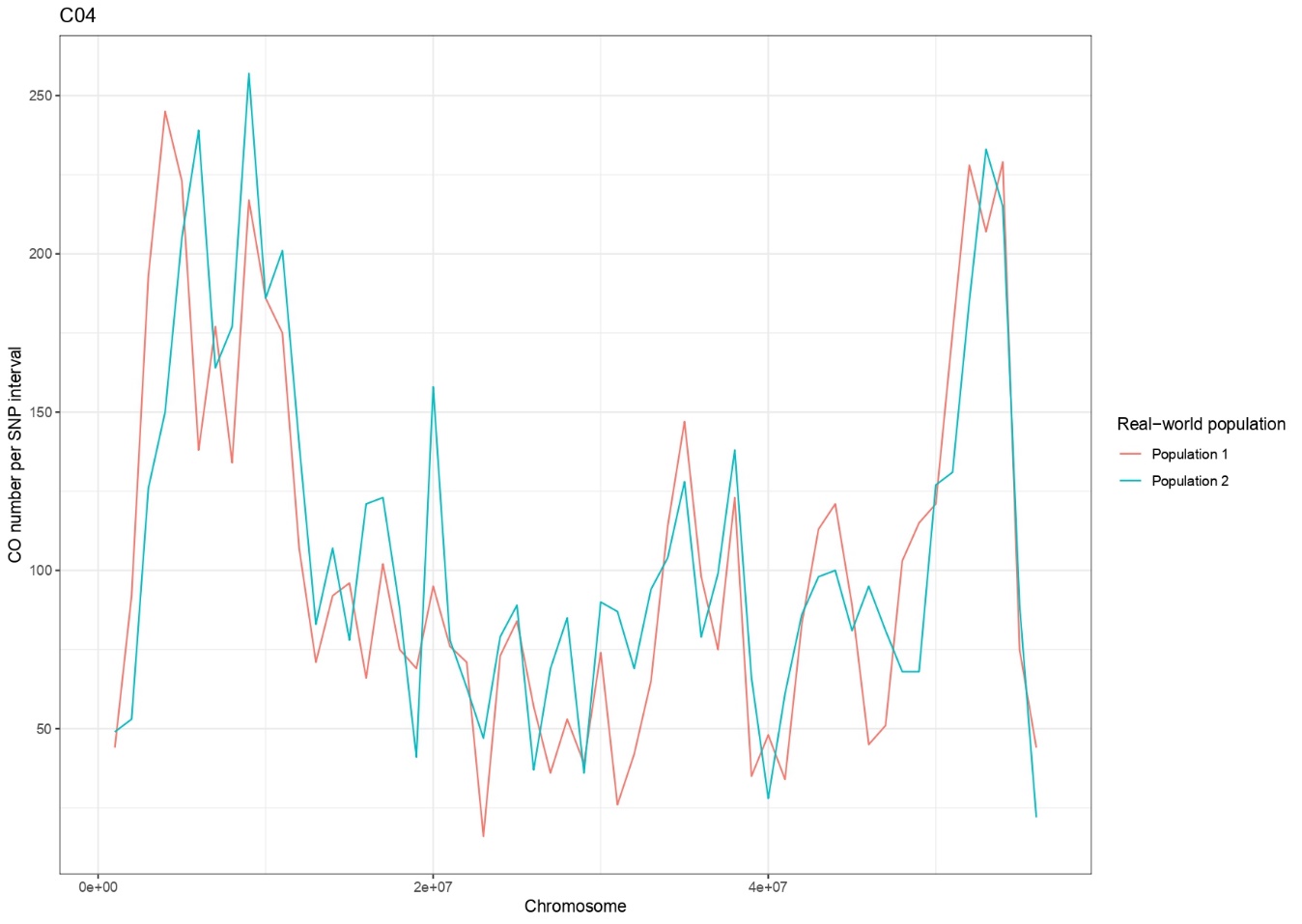

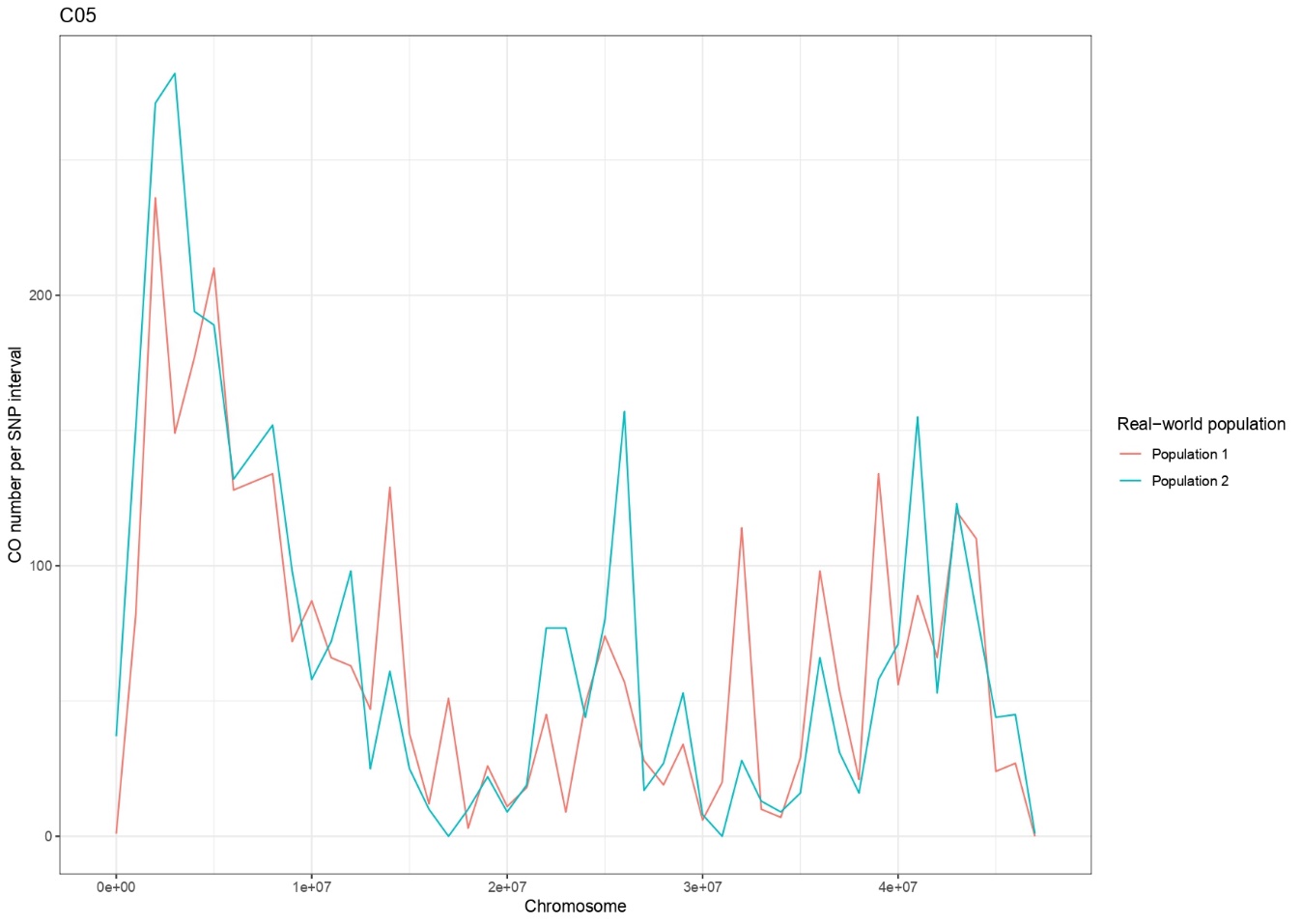

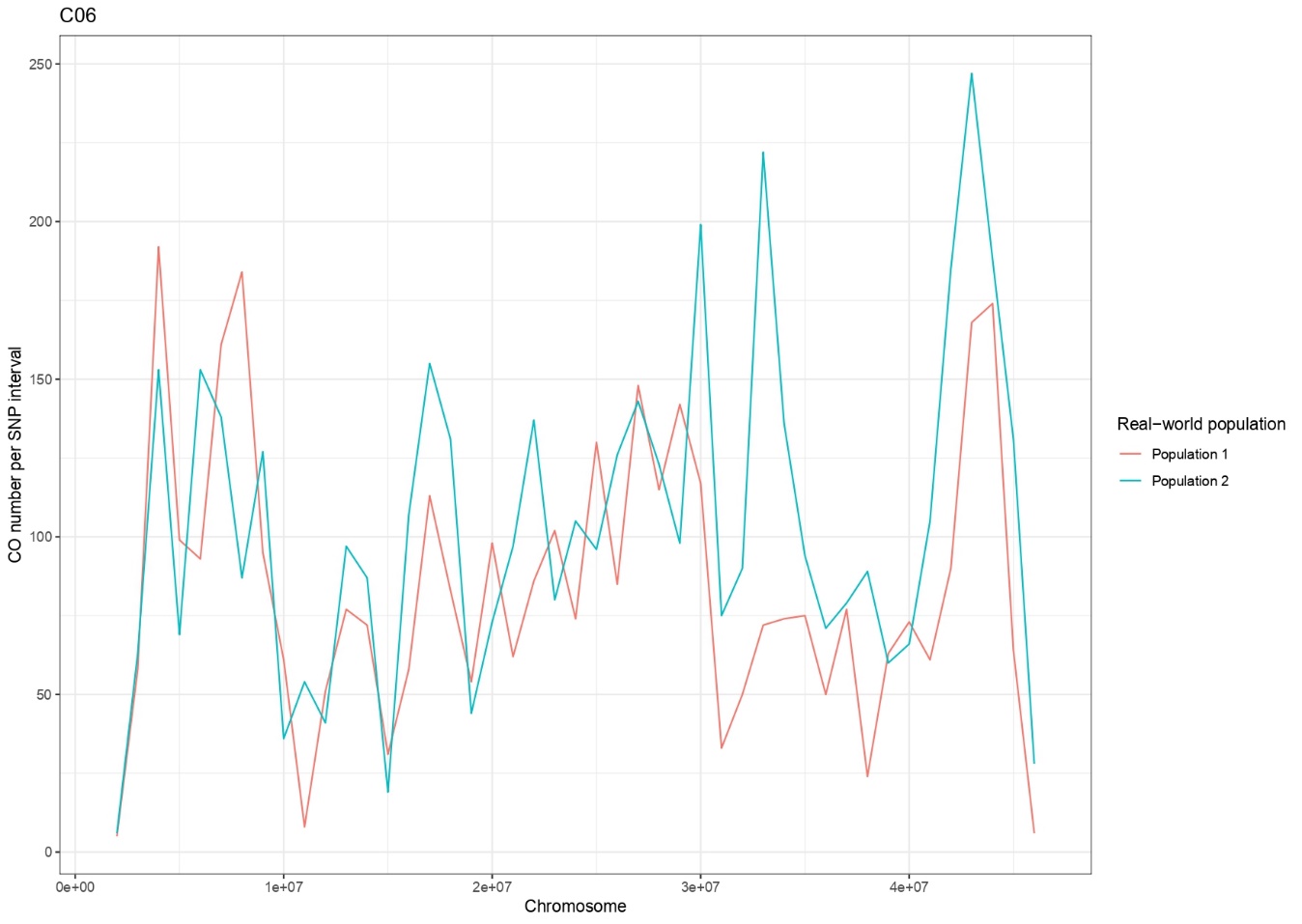

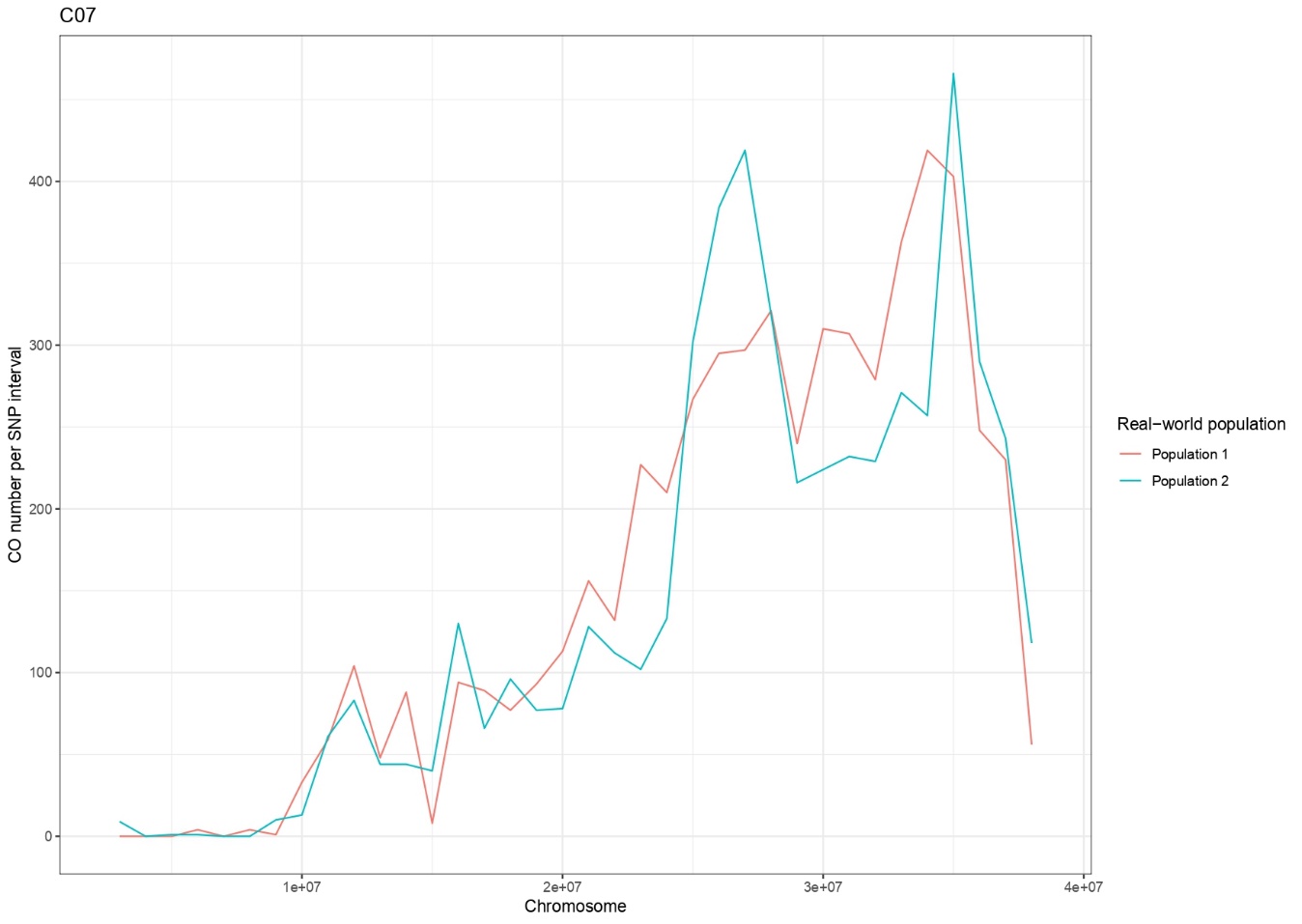

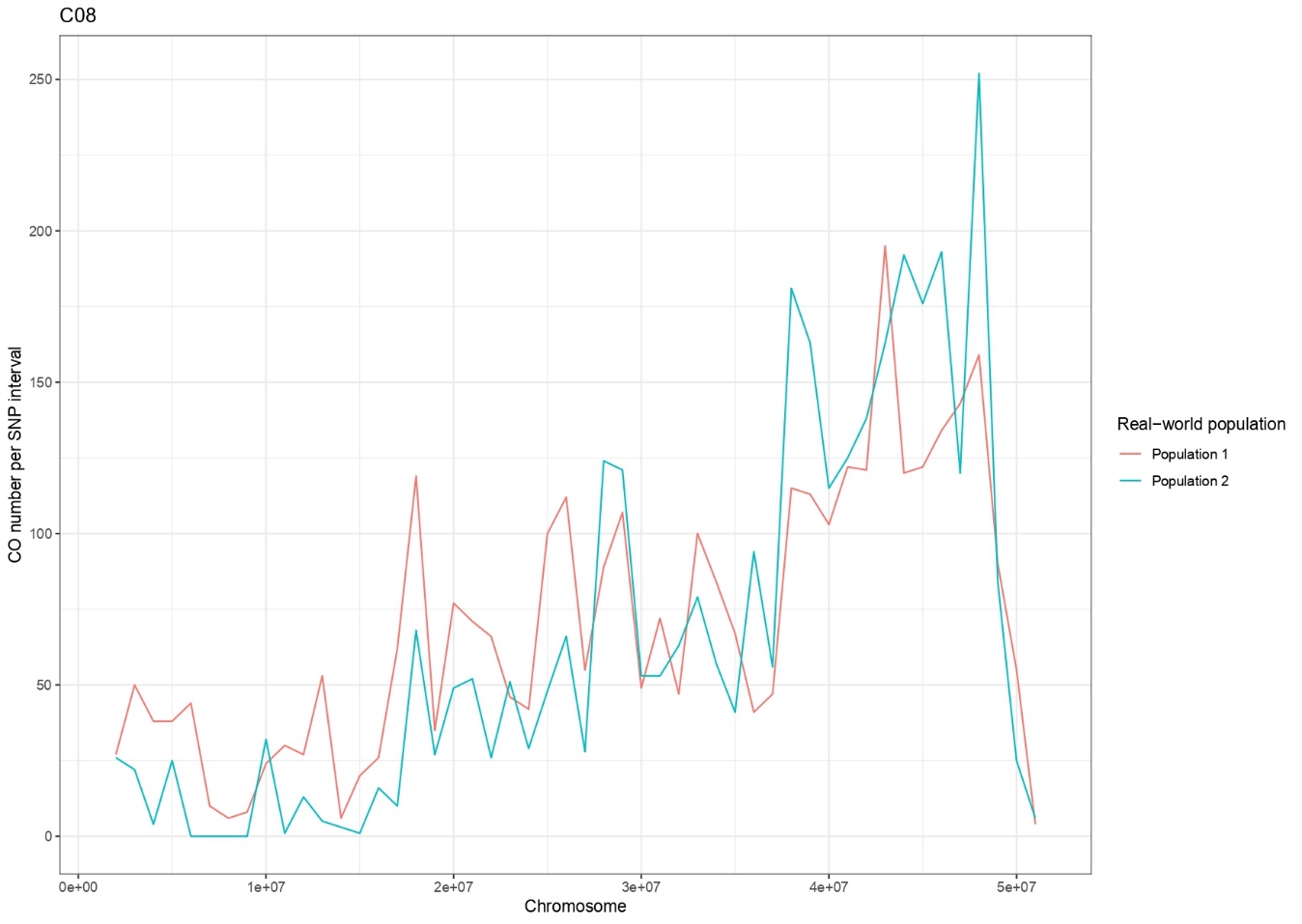

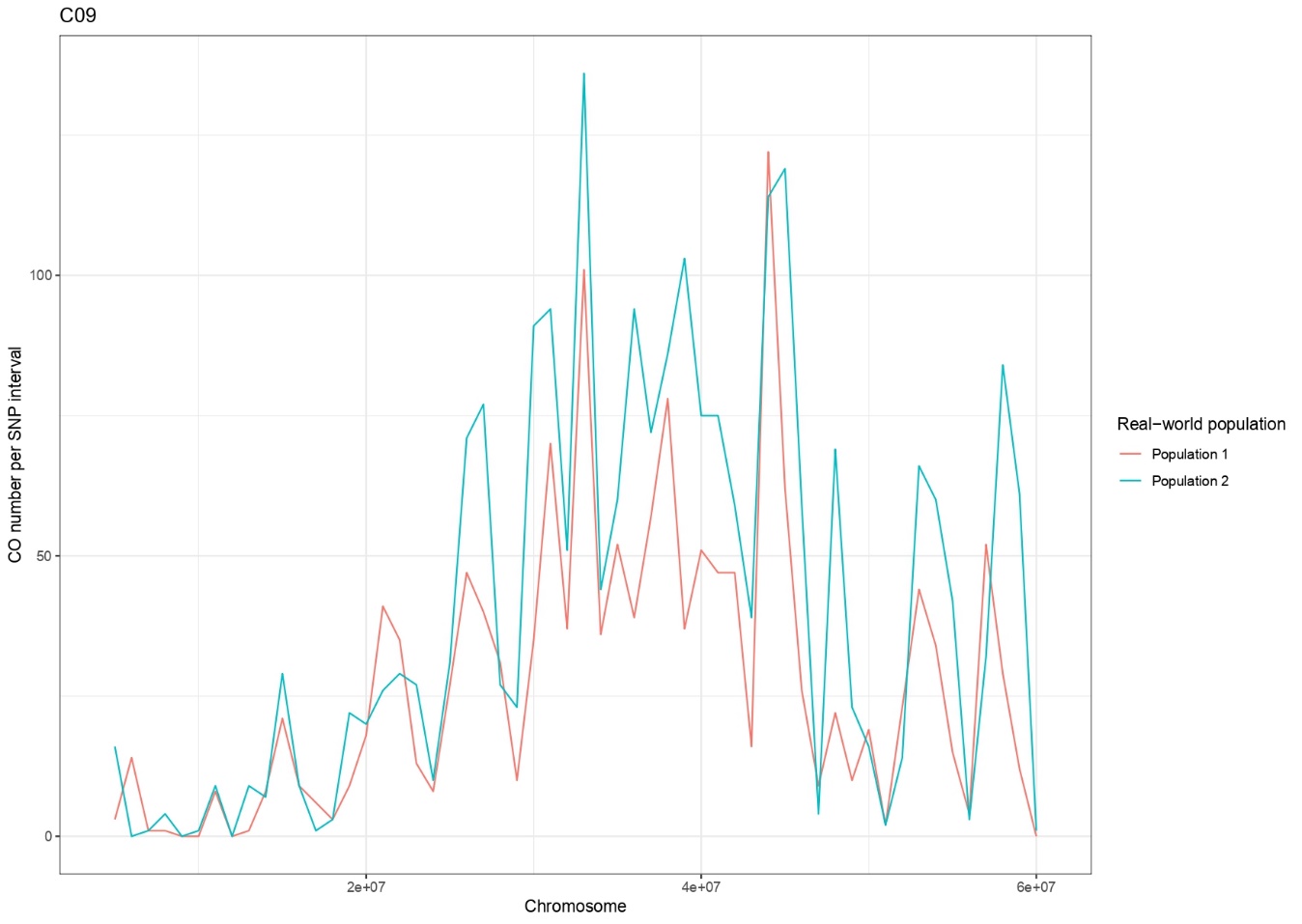


Figure S3 Distribution of the number of crossovers per SNP interval detected by haploMAGIC (min=2/5/3, imp=imputeTHonly, cor=correctFalseHom) and LINKPHASE3 (output from recombinations_hmm) in every informative meiosis across the 19 Brassica napus chromosomes, for which positions are indicated in base pairs. For each crossover gap, the measured numbers were normalized as the inverse of the number of SNP intervals within the gap, so that the crossover number in any recombination gap summed 1.


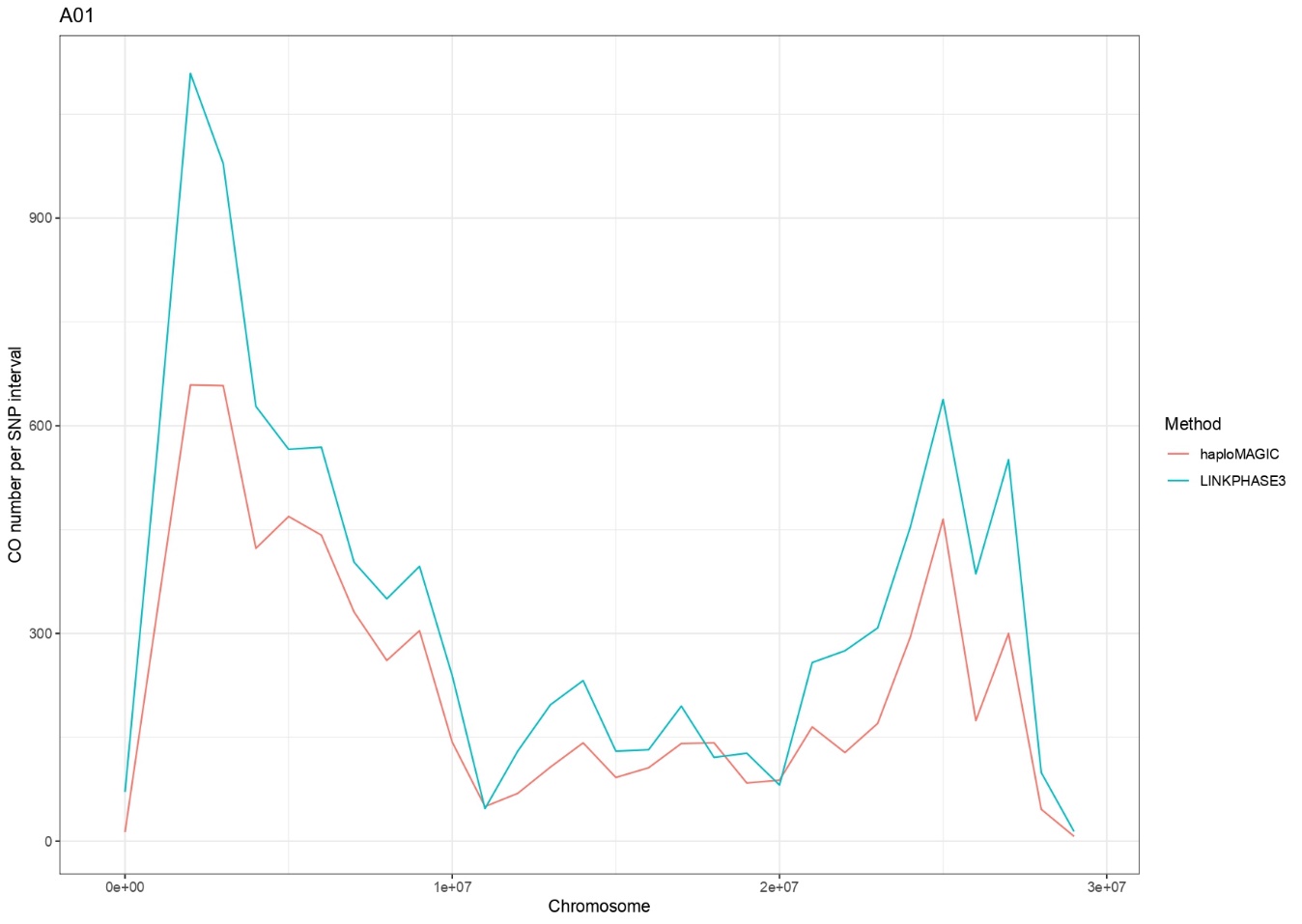


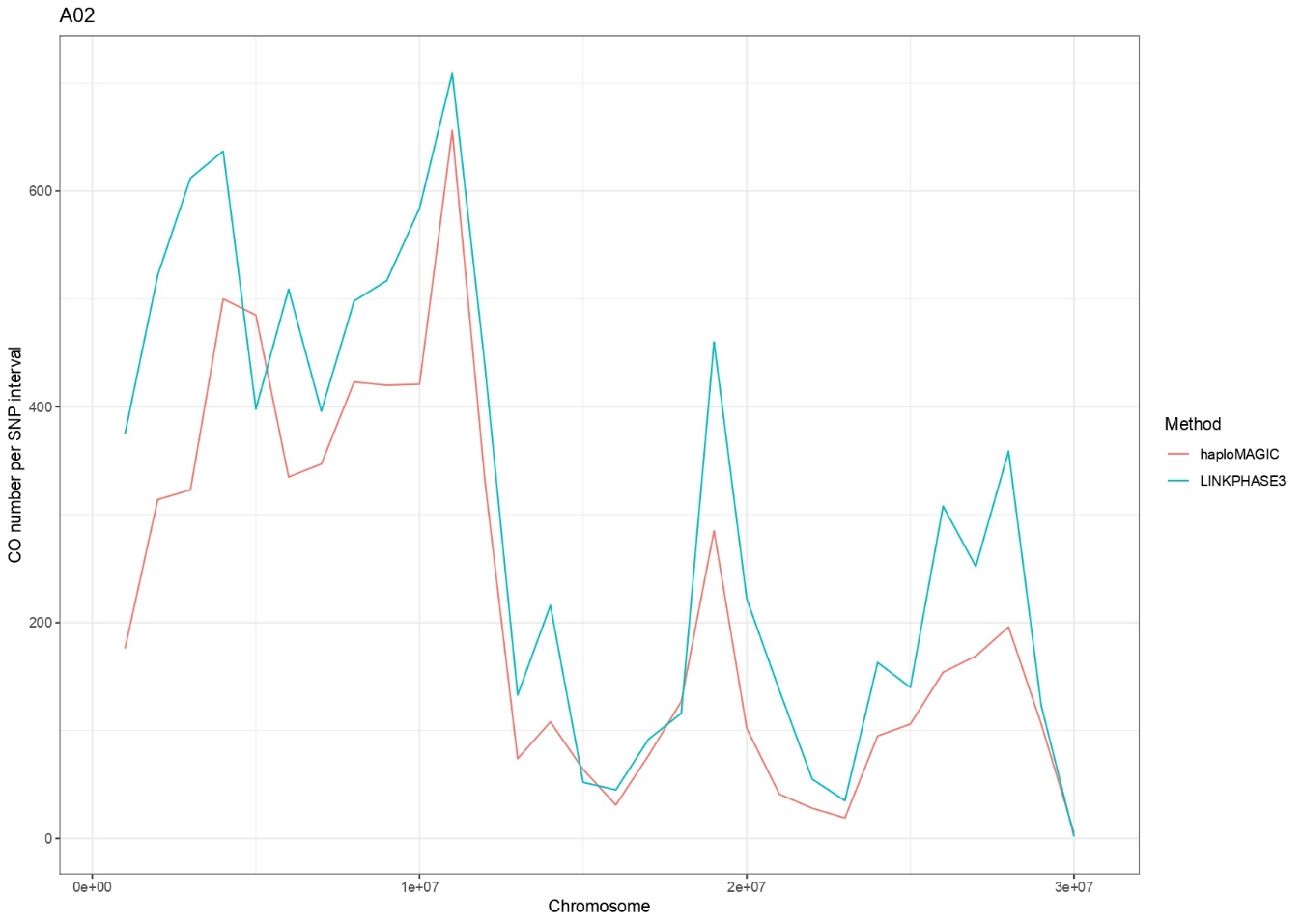

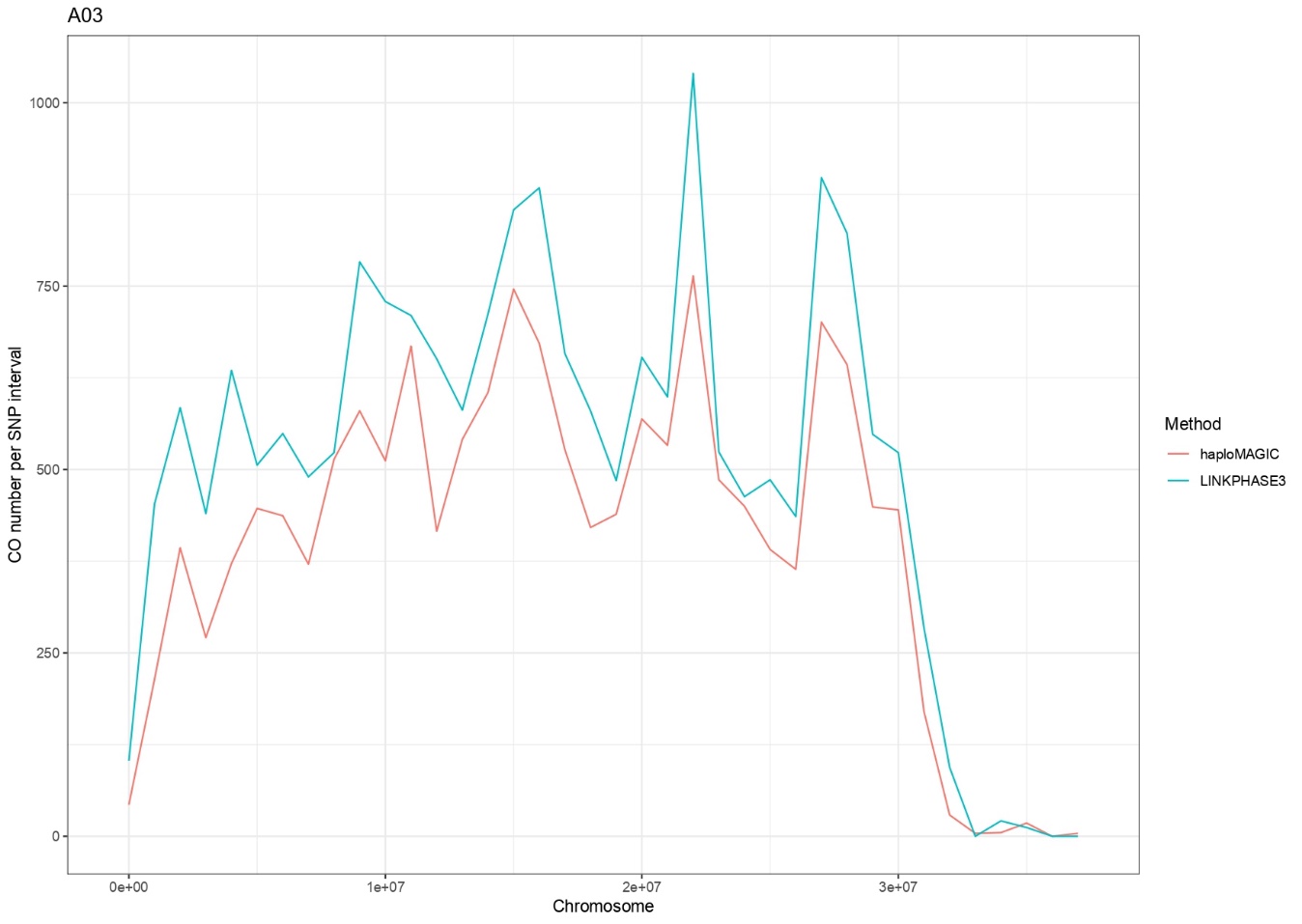

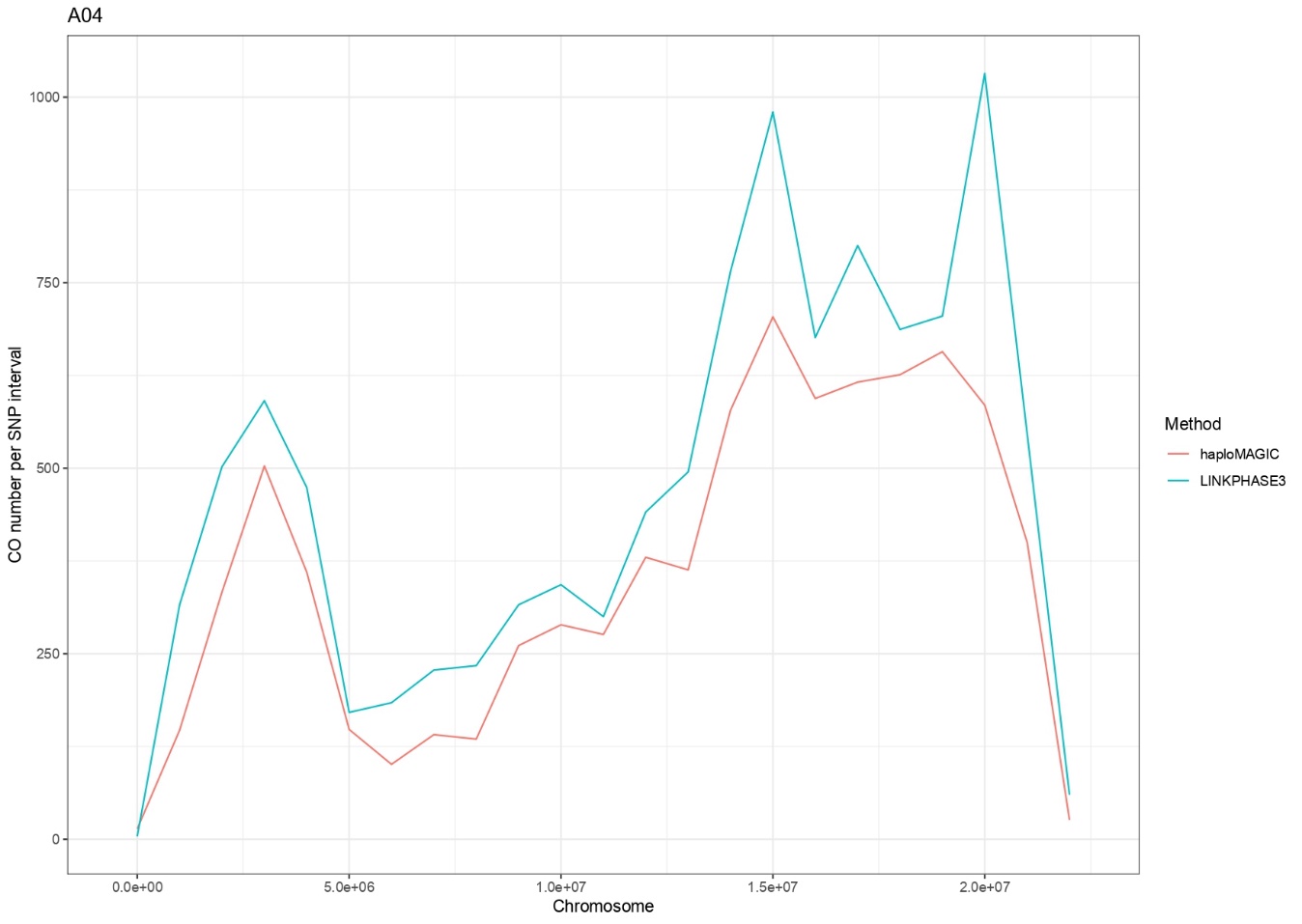

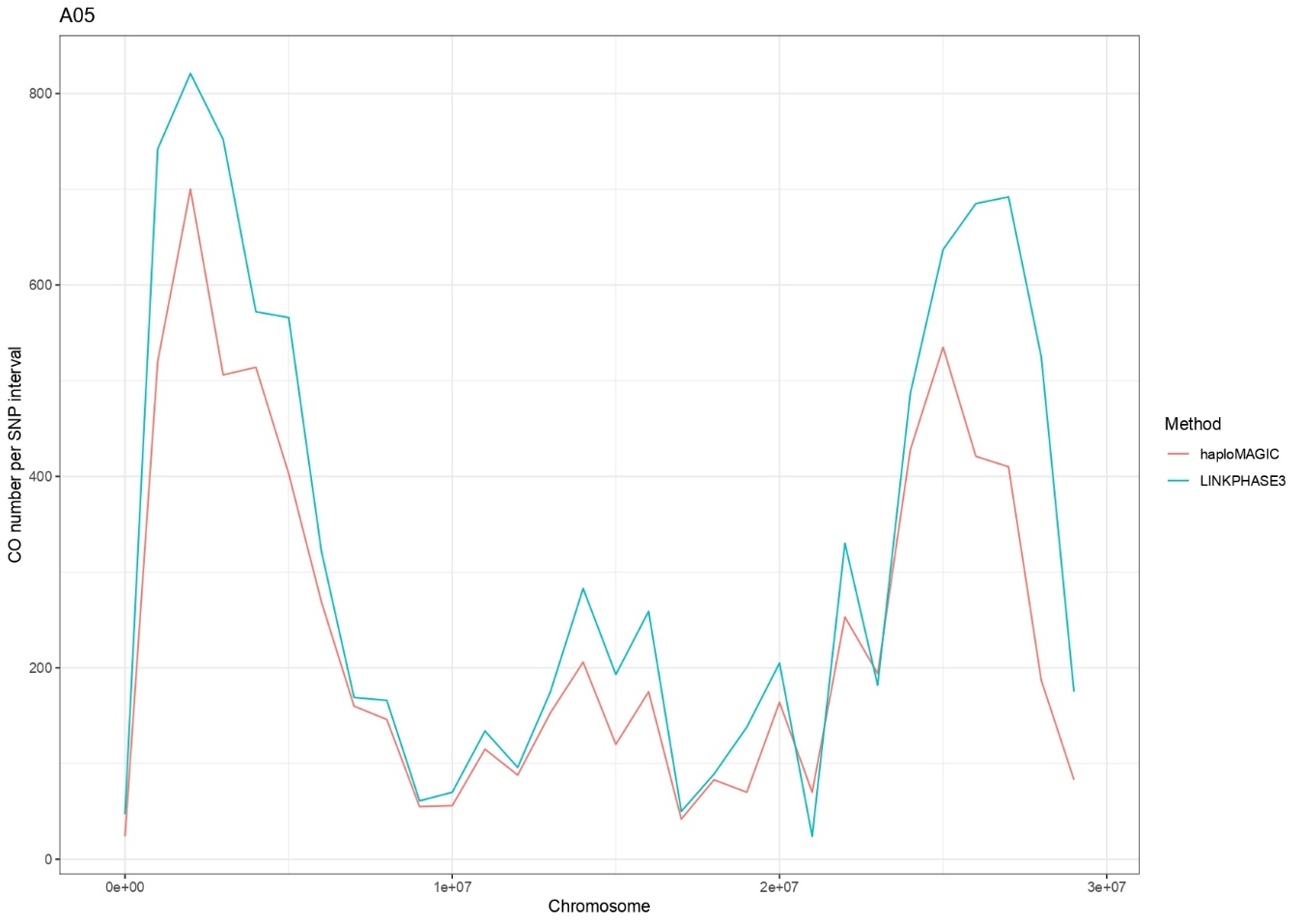

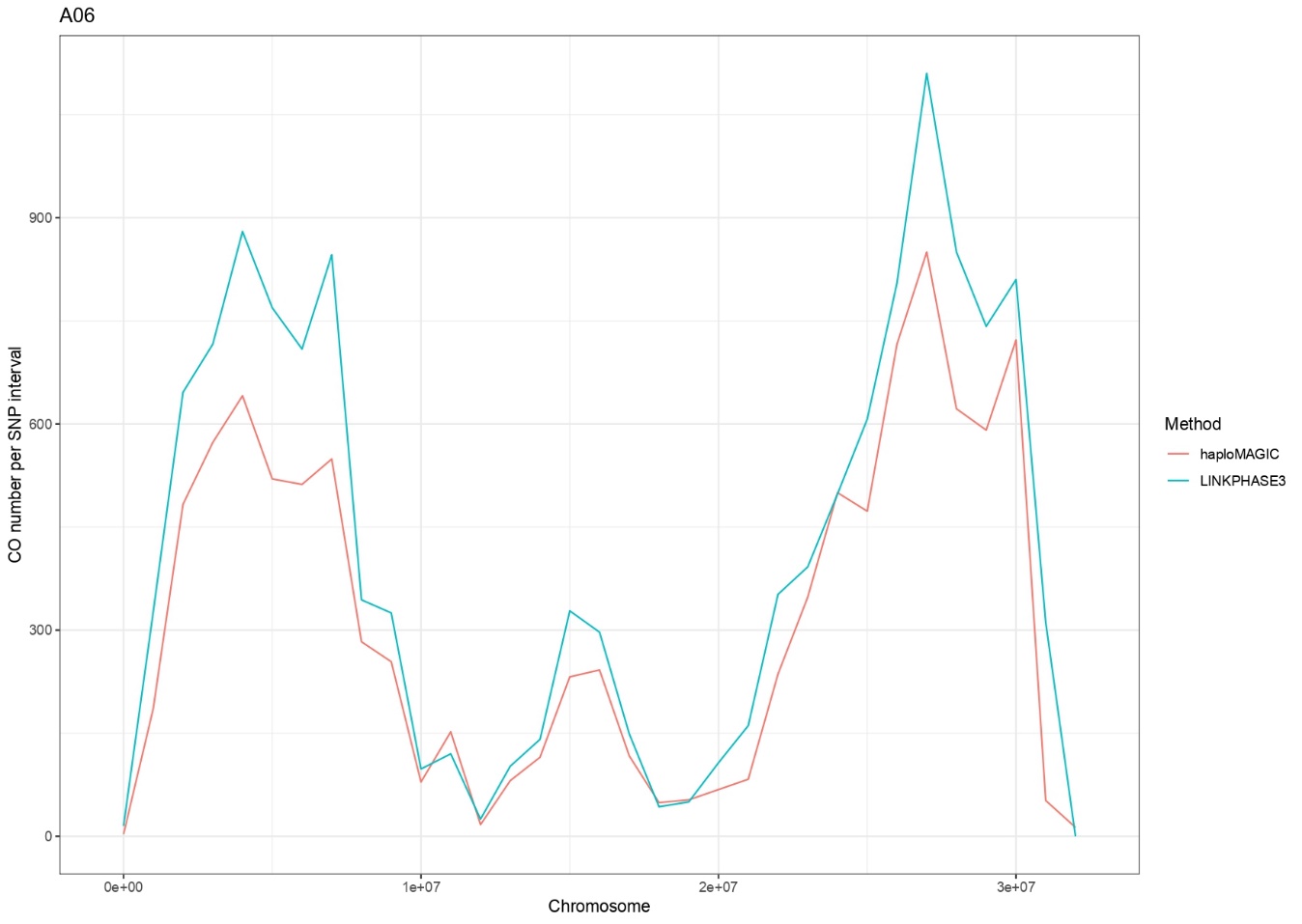

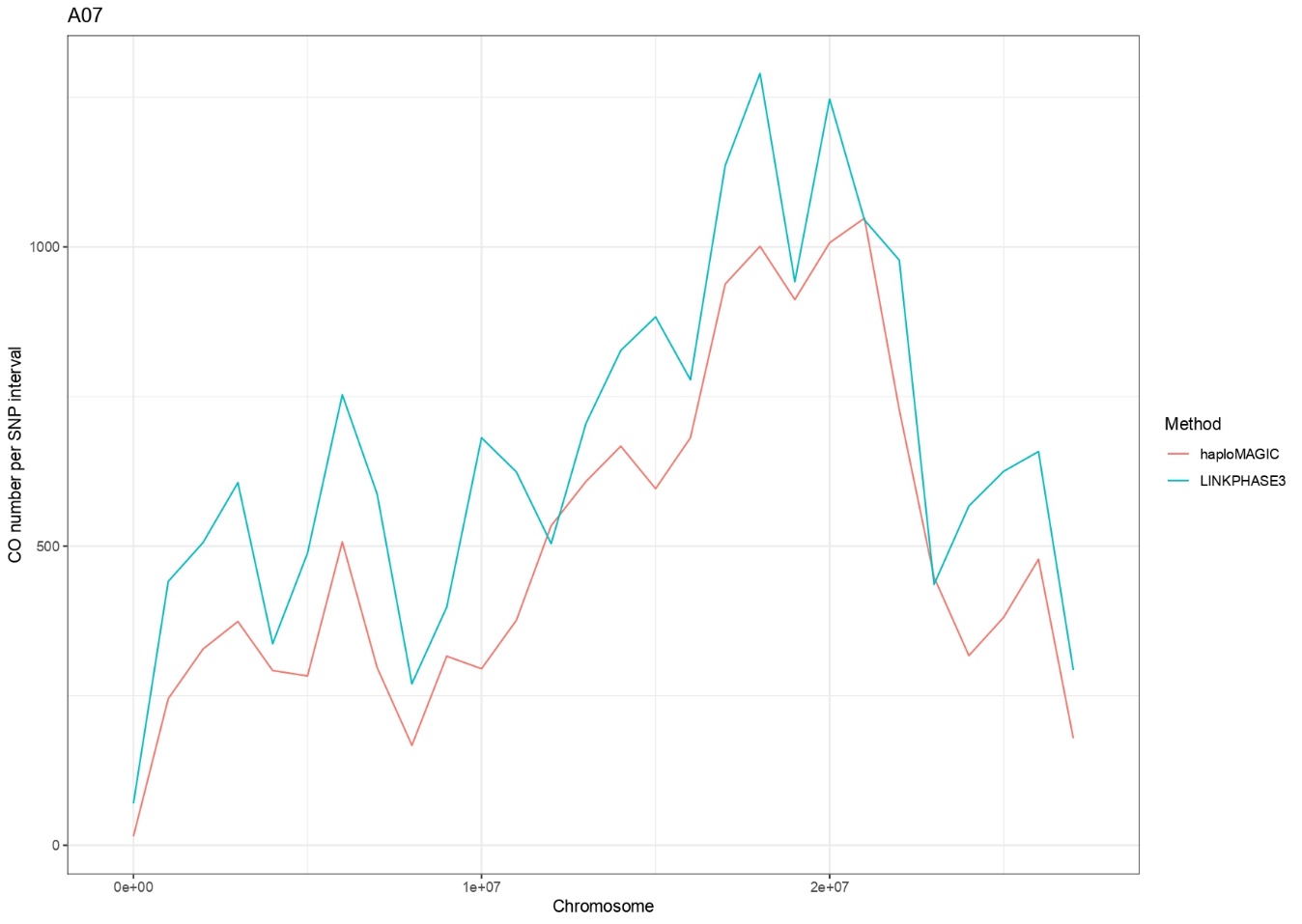

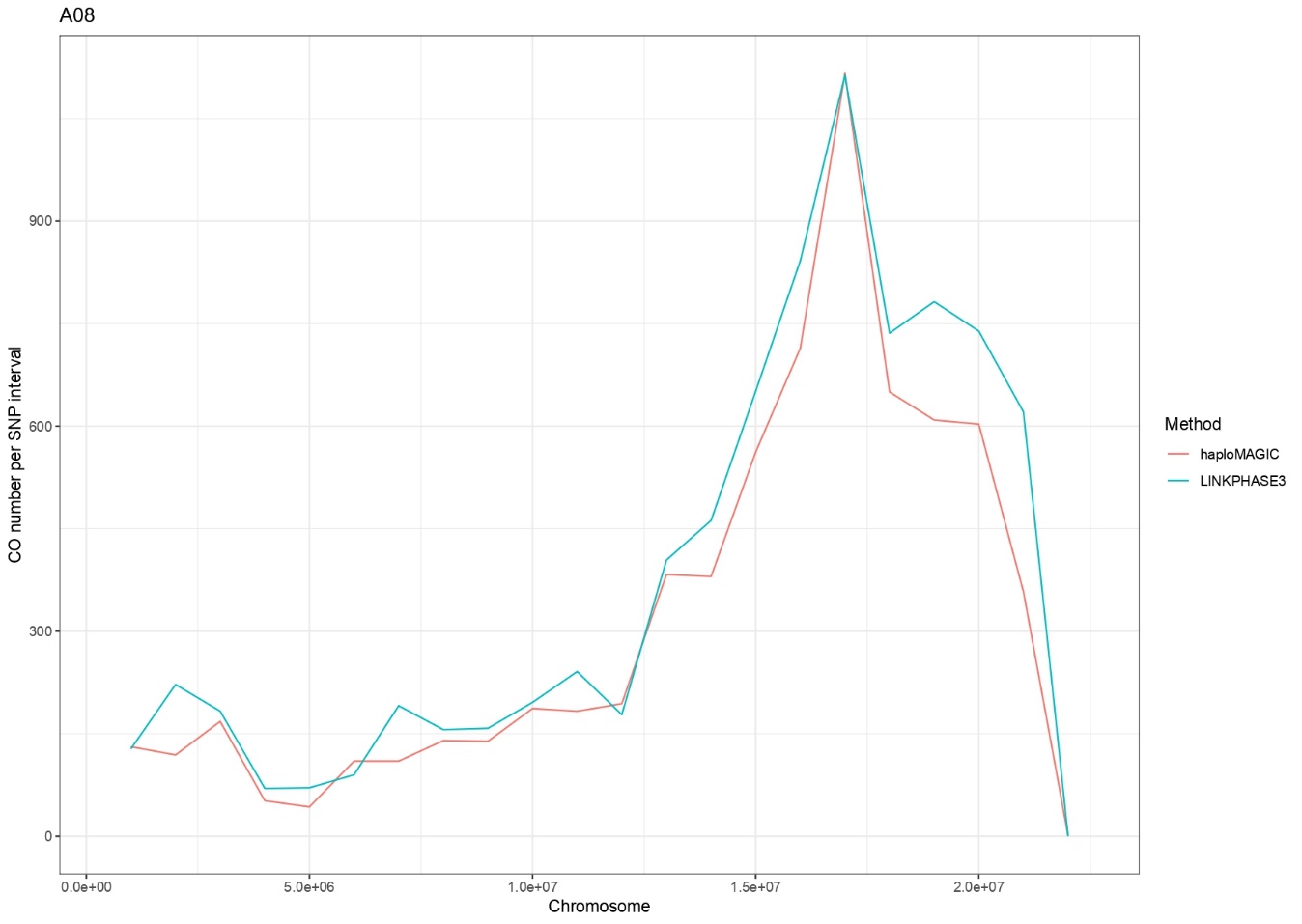

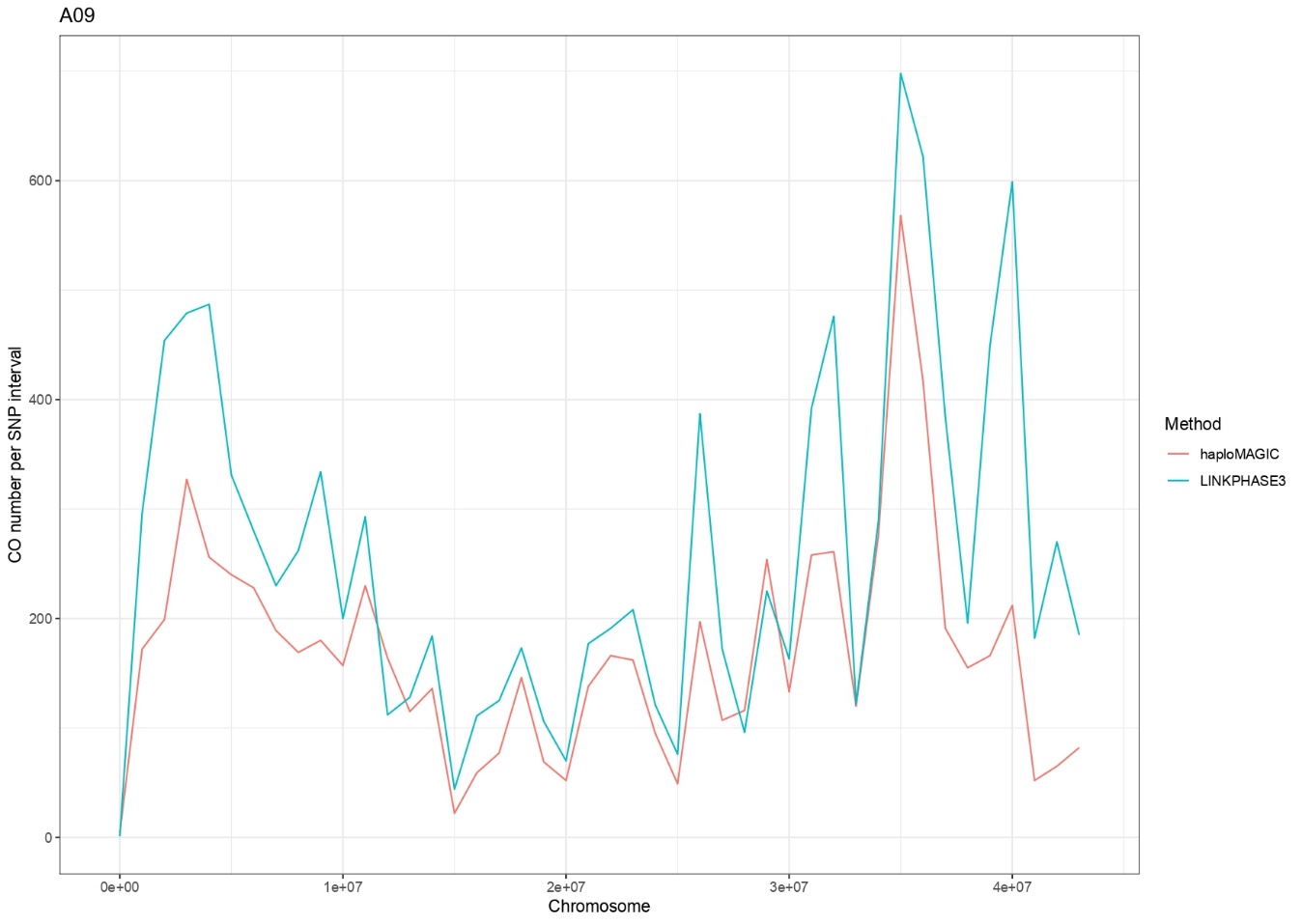

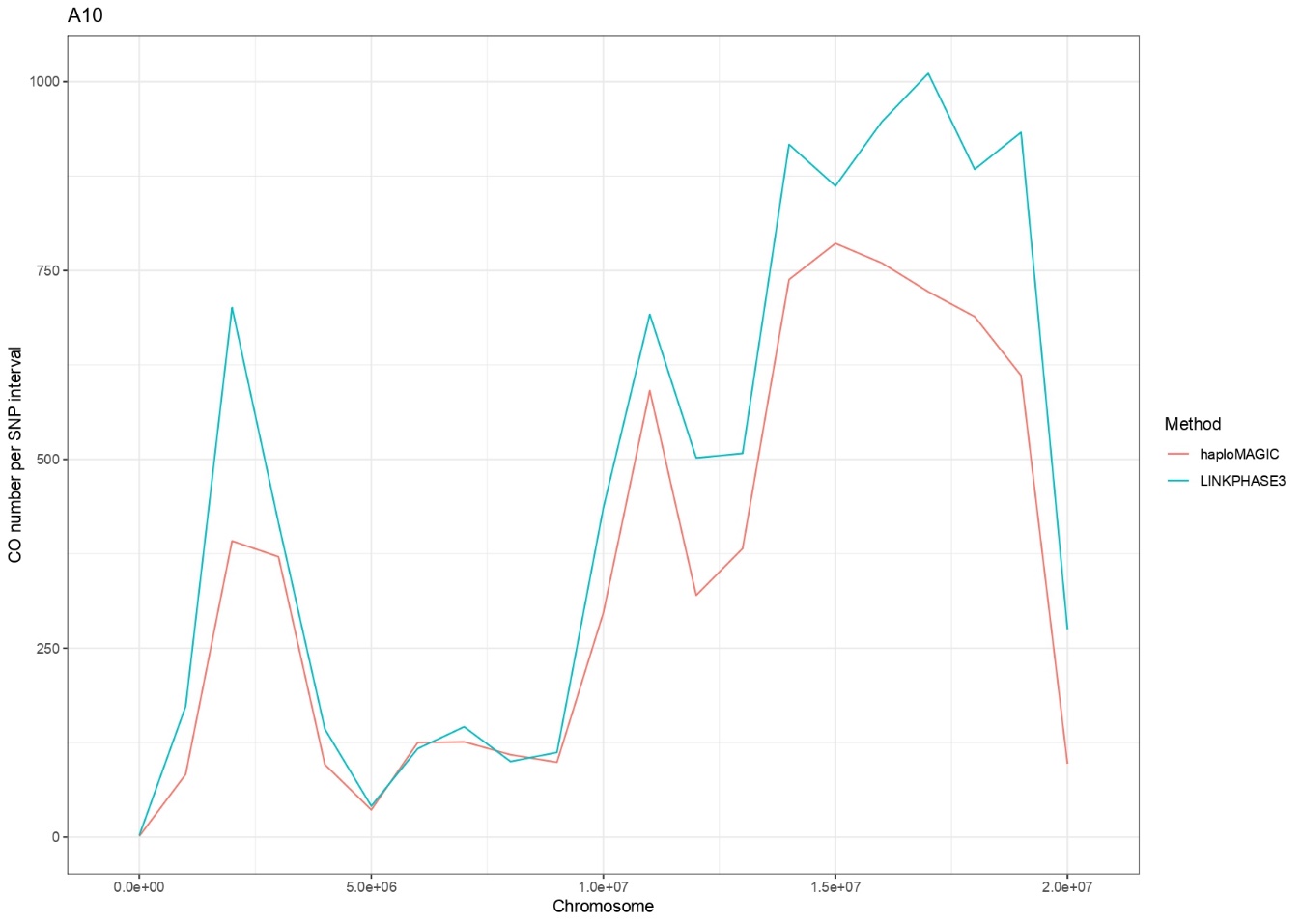

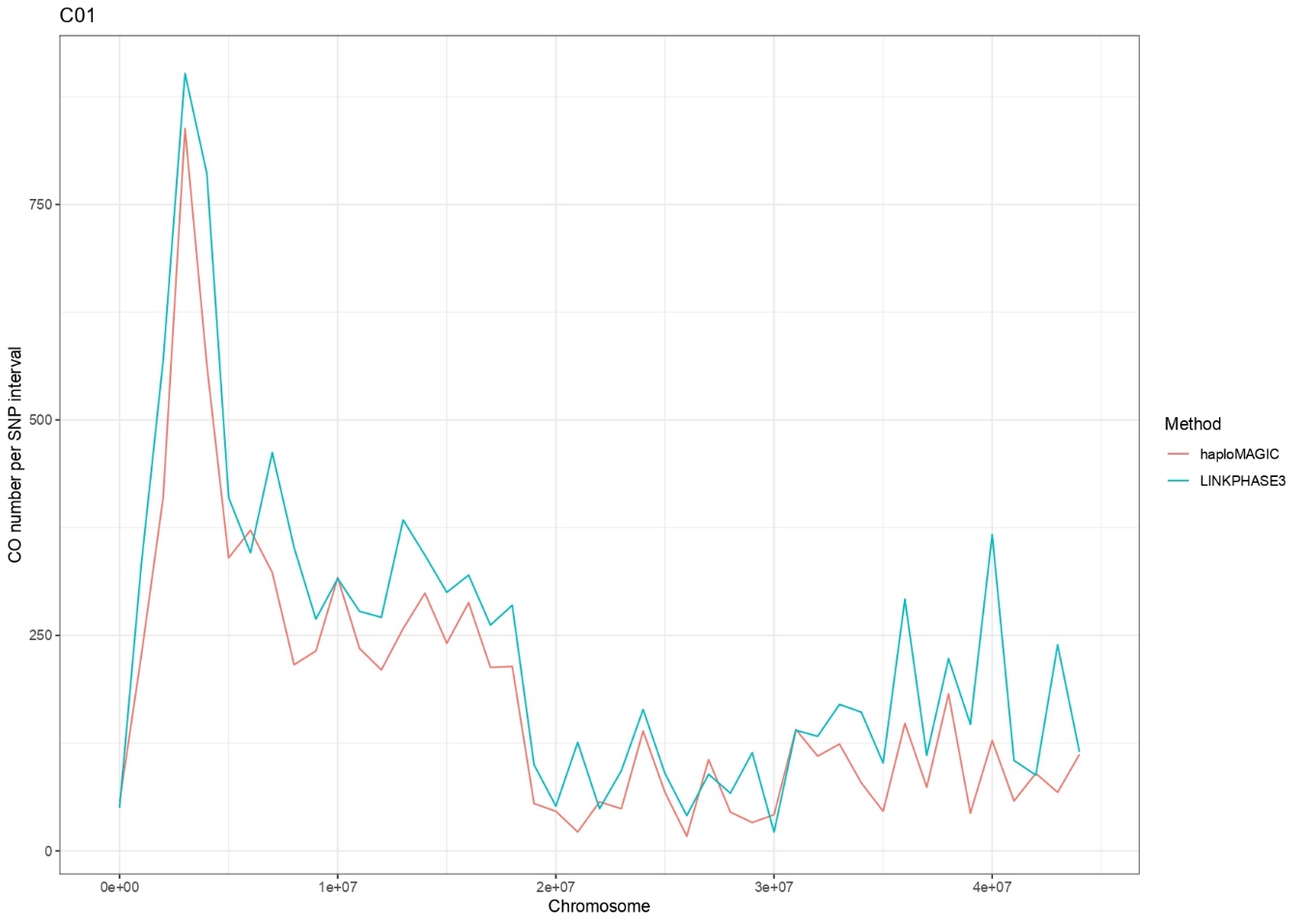

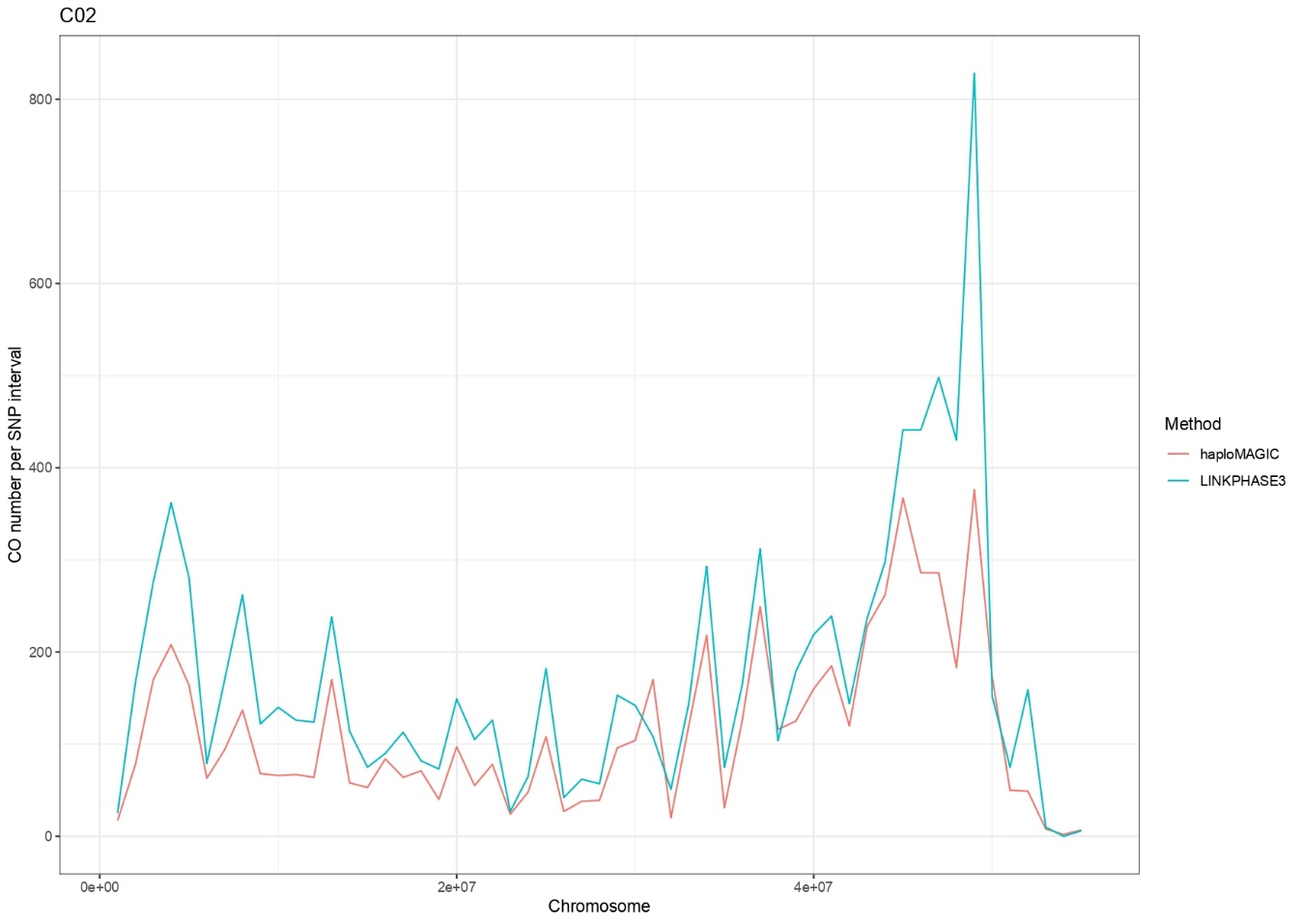

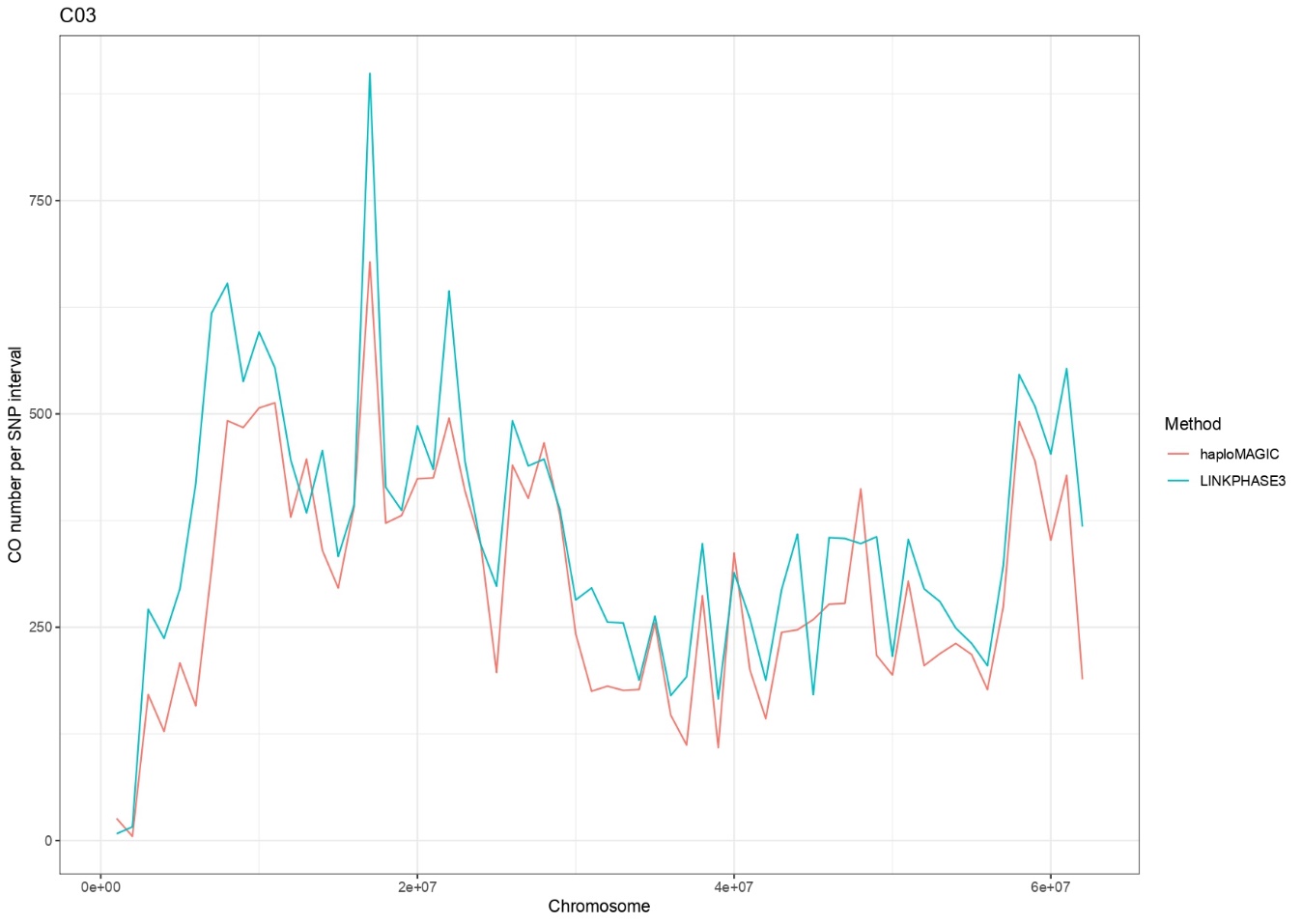

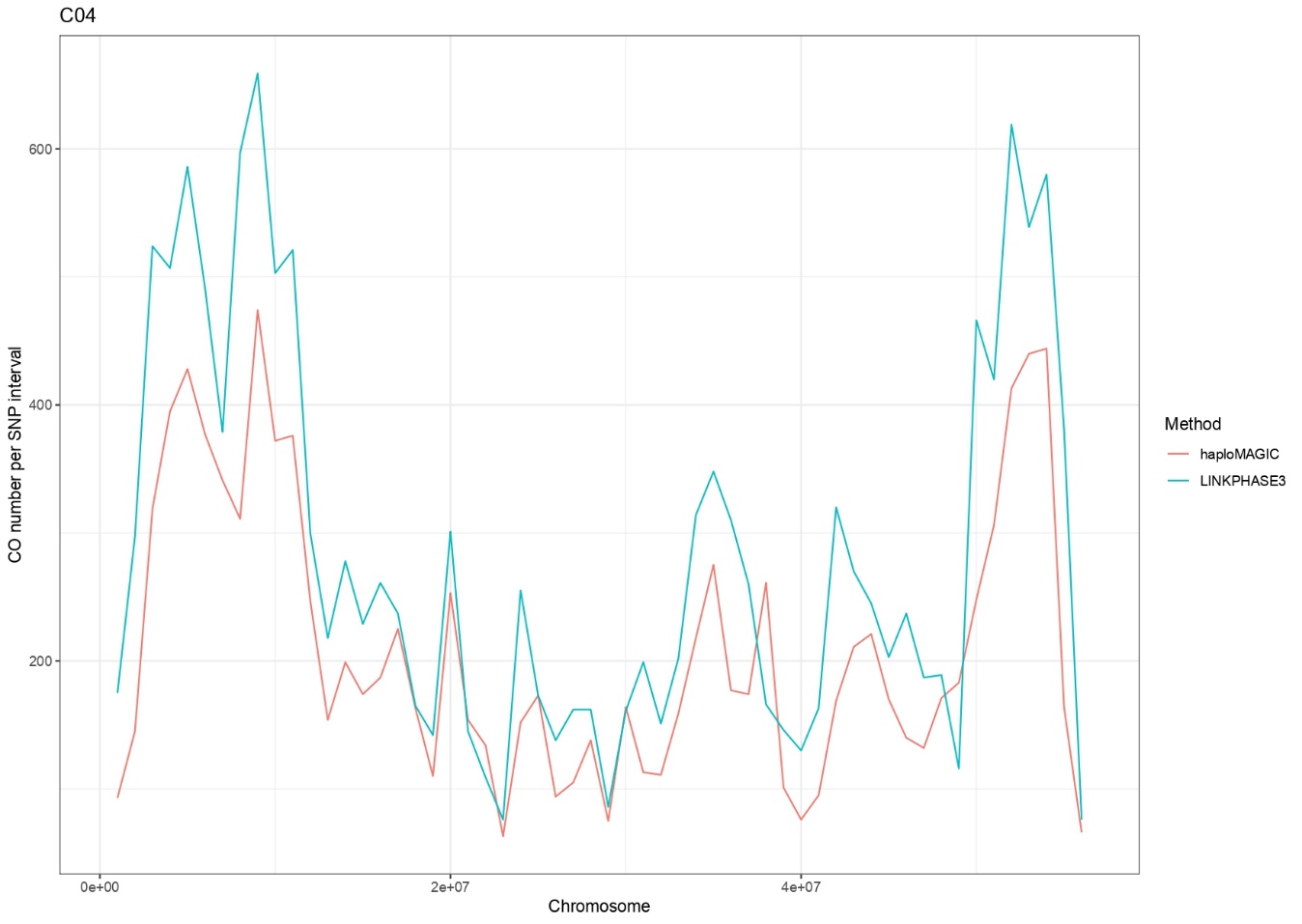

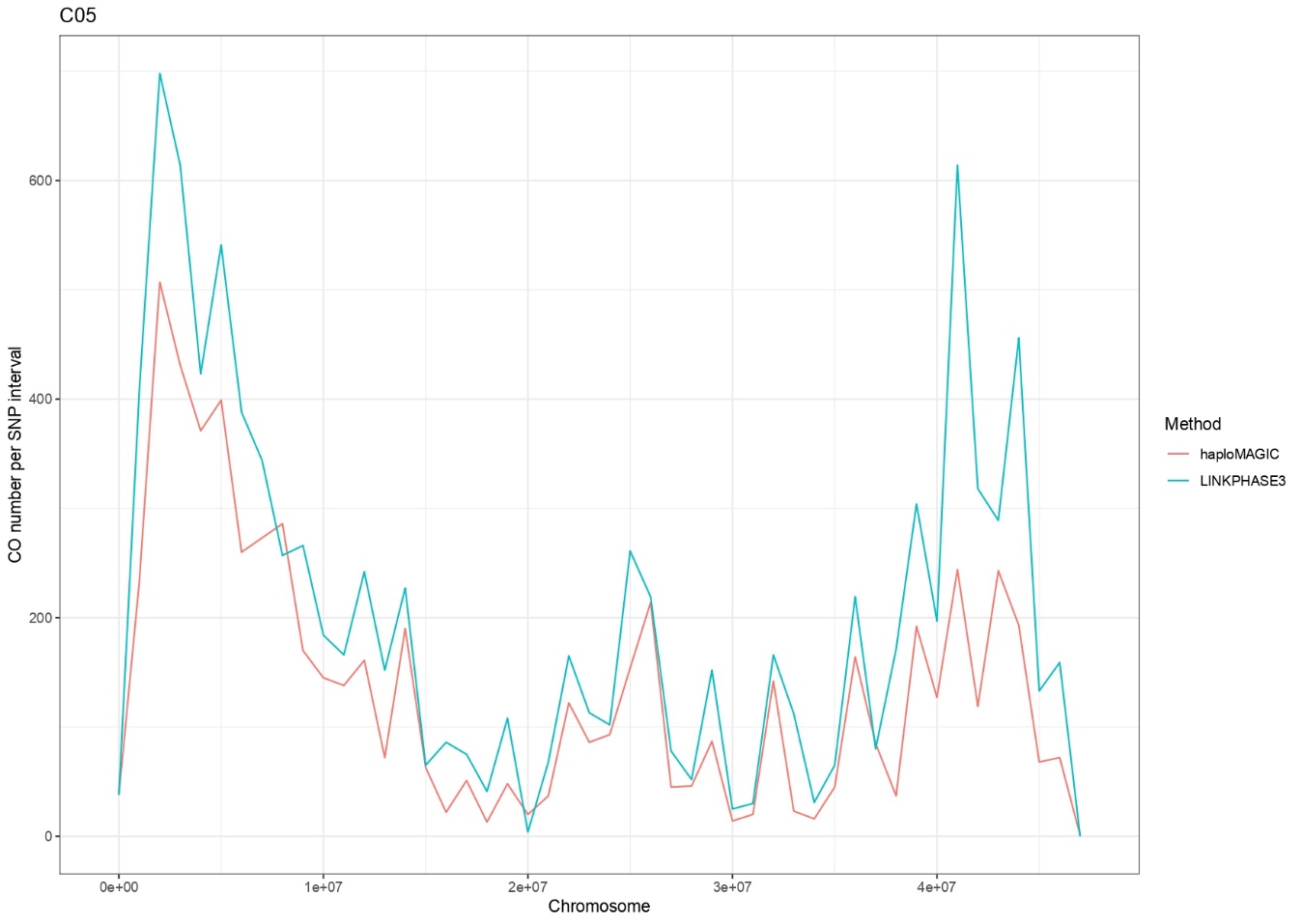

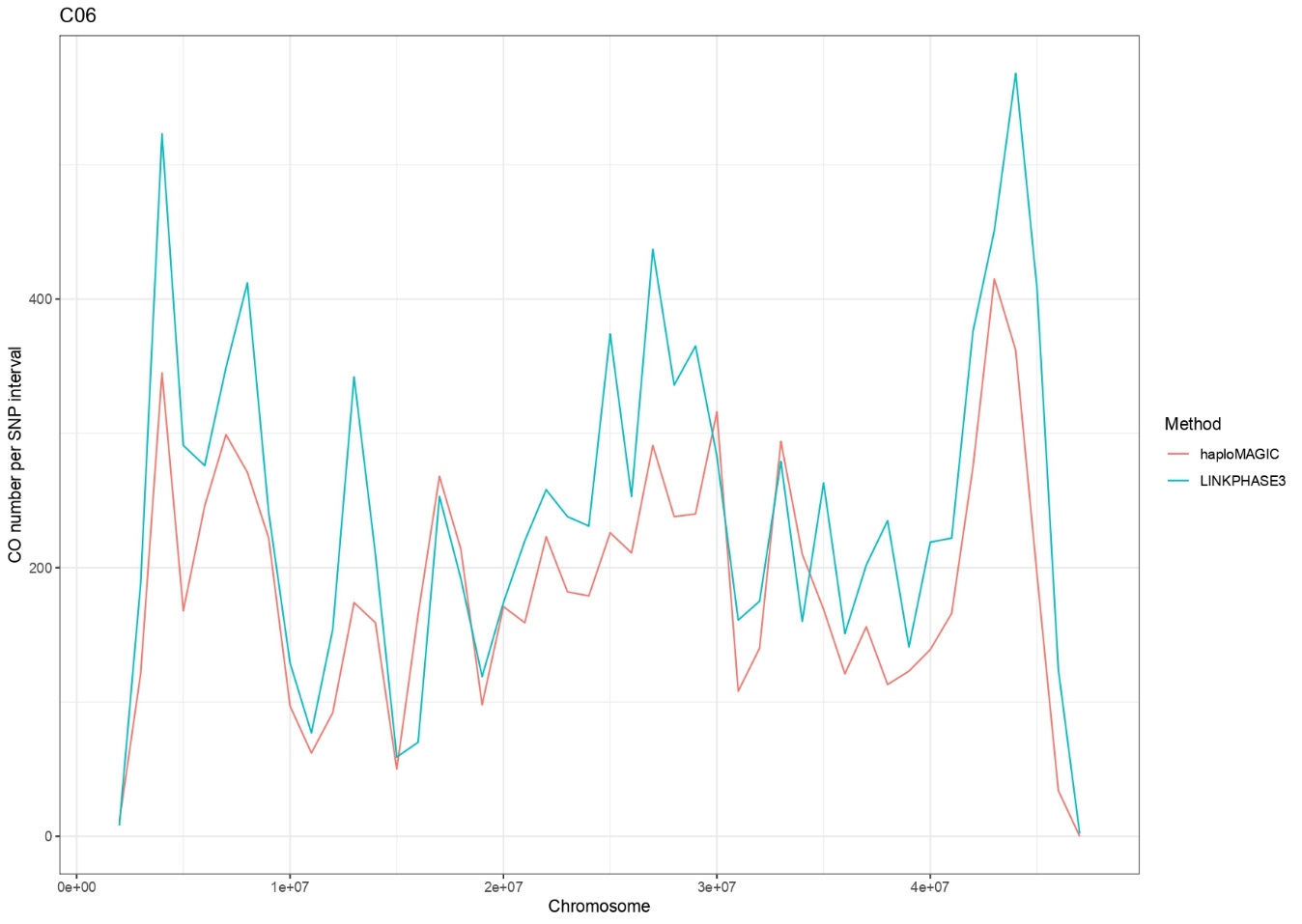

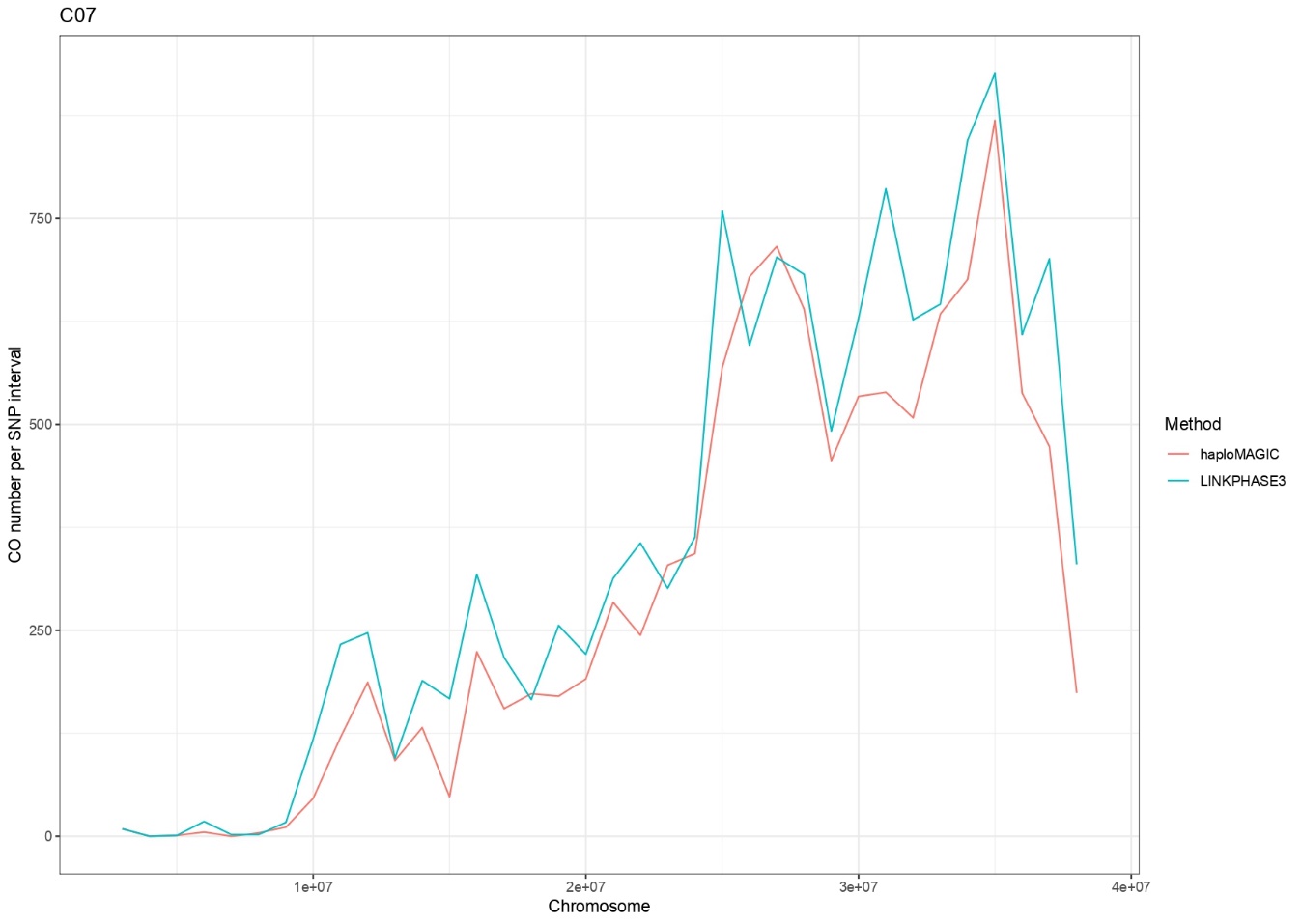

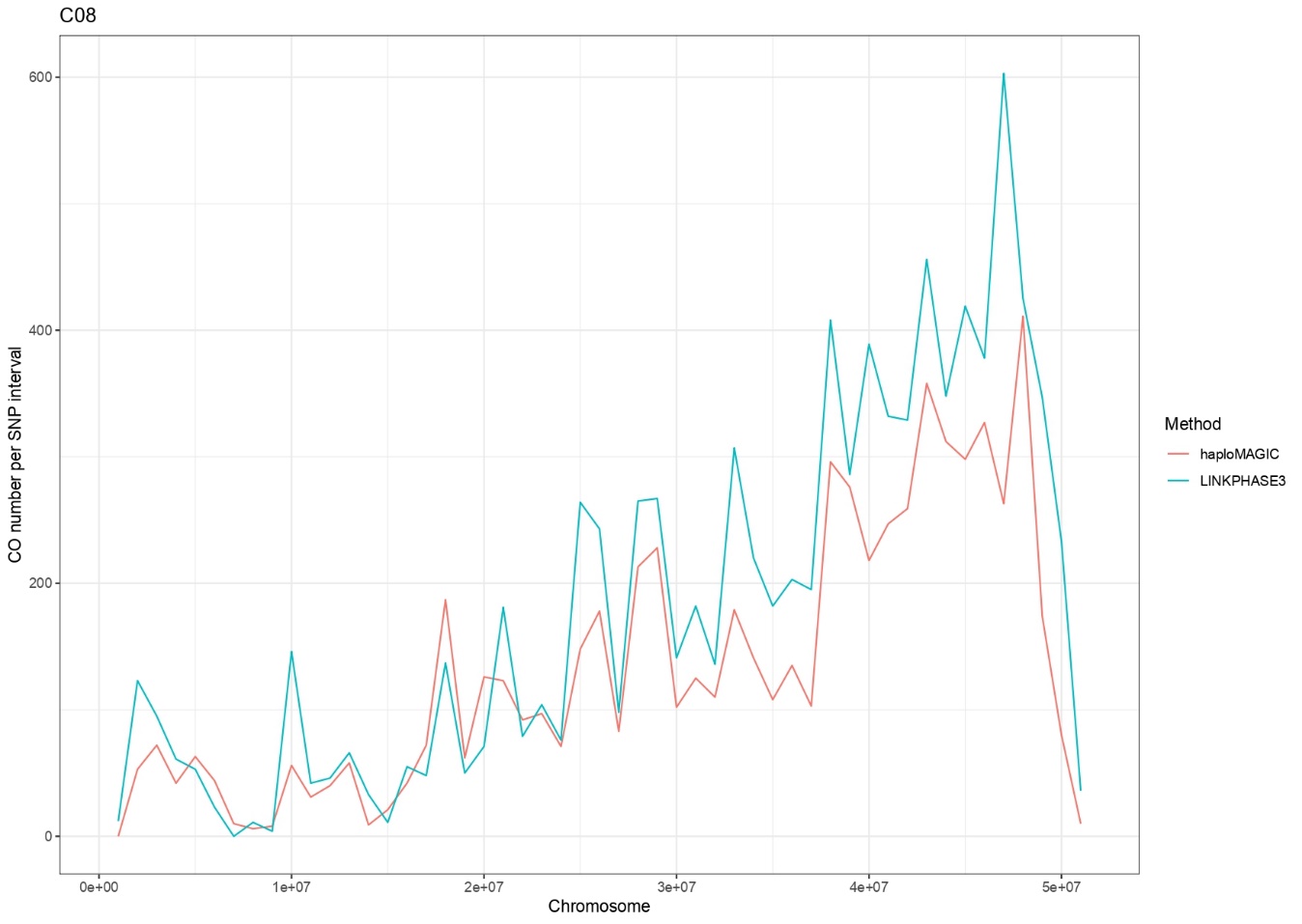

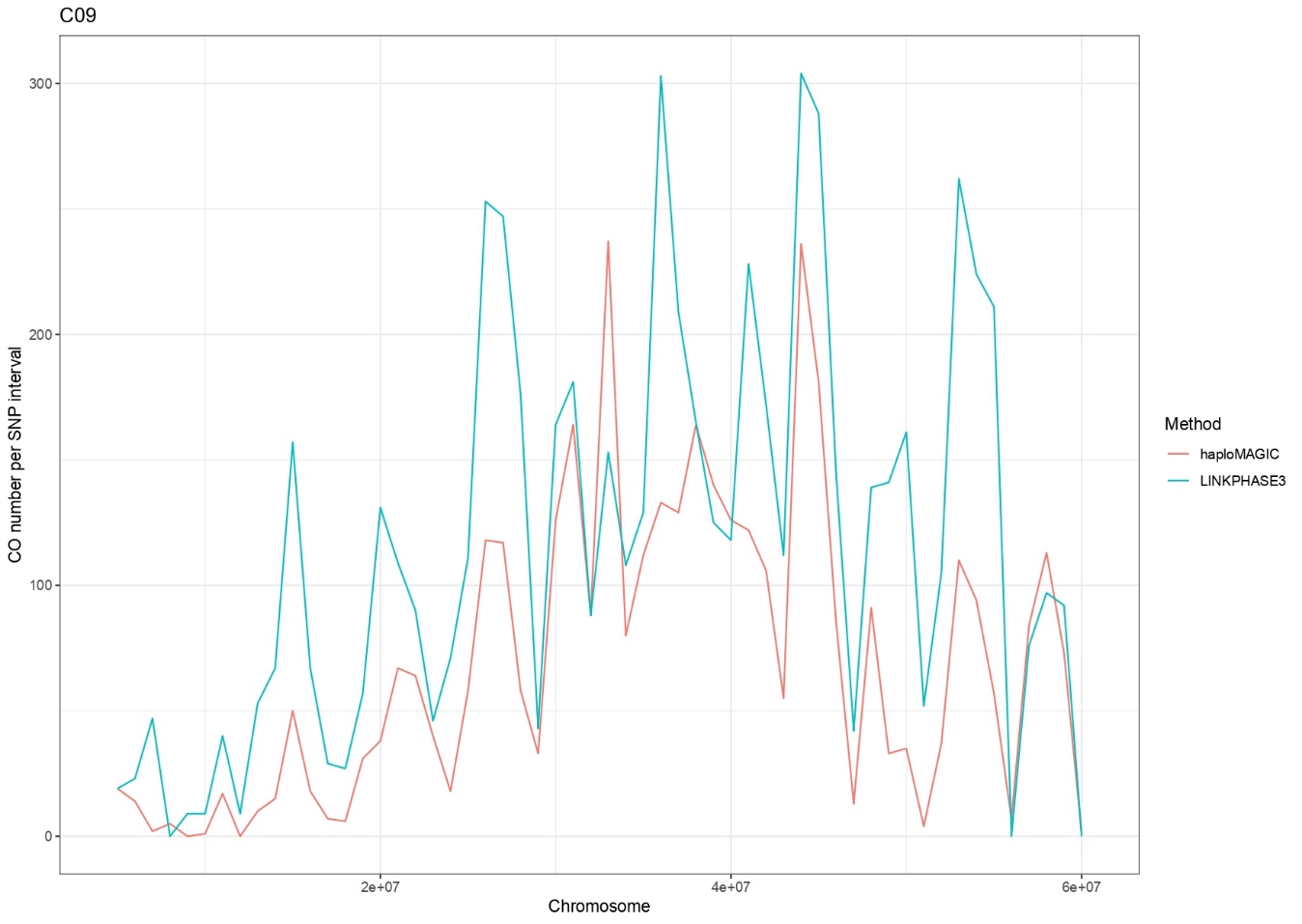

Supplement: jkae109_Supplementary_Data [file jkae109_supplementary_data.zip › Supplemental_Figures_G3-2024-405056.docx]
